# Supplementary material for: Intralesional steroid injections to prevent refractory strictures in patients with oesophageal atresia: study protocol for an international, multicentre randomised controlled trial (STEPS-EA trial)
Source: BMJ Open. 2019 Dec 16;9(12):e033030. doi: 10.1136/bmjopen-2019-033030 (PMC6937109; doi:10.1136/bmjopen-2019-033030)
Supplement: Supplementary data [file bmjopen-2019-033030supp001.pdf]

SUPPLEMENTARY FILE 1

To calculate the distribution of the number of dilatations in the steroid group, we assumed the following:

- The first 3 dilatations are unaffected by the intervention; thus the 4 patients with 3 dilatations in the observed data would still have 3 dilatations with steroid treatment.
- The 7 patients with 4 dilatations in the observed data would, if steroid treatment were applied, be equally divided over the categories 3 dilatations and 4 dilatations (3.5 patients in each category).
- The 9 patients with 5 dilatations in the observed data would, if steroid treatment were applied, have 4 dilatations.
- The 7 patients with 6 dilatations in the observed data would, if steroid treatment were applied, be equally divided over the categories 4 dilatations and 5 dilatations (3.5 patients in each category).
- The 16 patients with 7-10 dilatations in the observed data would, if steroid treatment were applied, be divided over the categories 5, 6 and 7-10 dilatations (patients with 7 dilatations would have 5 dilatations, patients with 8 dilatations would have 5 or 6 dilatations, patients with 9 dilatations would have 6 dilatations and patients with 10 dilatations would have 6 or 7 dilatations).
- The 10 patients with >10 dilatations in the observed data would, if steroid treatment were applied, be divided over the categories 7-10 and >10 dilatations (5 patients in each category).

These assumptions lead to the predicted numbers of patients shown in Supplementary Table 1. The relative frequency distribution in the steroid group is then calculated by dividing the predicted numbers of patients by the total number of 53 patients.

**Number of dilatations within 28 days interval**	**Observed number of patients (n=407) [1]**	**Predicted number of patients with steroid treatment**	**Relative frequencies control group**	**Assumed relative frequencies steroid group**
3 dilatations	4	7.5 (4+3.5)	0.075	0.142
4 dilatations	7	16 (3.5+9+3.5)	0.132	0.302
5 dilatations	9	9 (3.5+2+3.5)	0.170	0.170
6 dilatations	7	8.5 (3.5+3+2)	0.132	0.160
7-10 dilatations*	16	7 (2+5)	0.302	0.132
>10 dilatations*	10	5 (5)	0.189	0.094
Total (all numbers of dilatations combined)	53	53	1.000	1.000

Supplementary Table 1: Assumed relative frequencies of the number of dilatations in the control and steroid groups, including the calculated predicted number of patients with steroid treatment. *See Supplementary Table 2 for the exact number of dilatations within these categories.

**Number of dilatations within 28 days interval**	**Observed number of patients (n=407)[1]**
3 dilatations	4
4 dilatations	7
5 dilatations	9
6 dilatations	7
7 dilatations	2
8 dilatations	7
9 dilatations	3
10 dilatations	4
12 dilatations	2
13 dilatations	2
15 dilatations	1
18 dilatations	2
24 dilatations	1
30 dilatations	1
34 dilatations	1
Total (all numbers of dilatations combined)	53

Supplementary Table 2: *Exact* number of dilatations as extracted from the *original dataset* of the retrospective study in the Netherlands.[1]

1. Vergouwe FWT, Vlot J, H IJ, et al. Risk factors for refractory anastomotic strictures after oesophageal atresia repair: a multicentre study. *Arch Dis Child* 2018.
