# Supplementary material for: Intralesional steroid injections to prevent refractory strictures in patients with oesophageal atresia: study protocol for an international, multicentre randomised controlled trial (STEPS-EA trial)
Source: BMJ Open. 2019 Dec 16;9(12):e033030. doi: 10.1136/bmjopen-2019-033030 (PMC6937109; doi:10.1136/bmjopen-2019-033030)
Supplement: Author's manuscript [file bmjopen-2019-033030.draft_revisions.pdf]

BMJ Open is committed to open peer review. As part of this commitment we make the peer review history of every article we publish publicly available.

When an article is published we post the peer reviewers' comments and the authors' responses online. We also post the versions of the paper that were used during peer review. These are the versions that the peer review comments apply to.

The versions of the paper that follow are the versions that were submitted during the peer review process. They are not the versions of record or the final published versions. They should not be cited or distributed as the published version of this manuscript.

BMJ Open is an open access journal and the full, final, typeset and author-corrected version of record of the manuscript is available on our site with no access controls, subscription charges or pay-per-view fees (http://bmjopen.bmj.com).

If you have any questions on BMJ Open's open peer review process please email info.bmjopen@bmj.com

BMJ Open

# The STEPS-EA trial: intralesional STEroid injections to Prevent refractory Strictures in patients with Esophageal Atresia – study protocol for an international, multicentre randomised controlled trial



| | |
|---|---|
| Journal: | *BMJ Open* |
| Manuscript ID | bmjopen-2019-033030 |
| Article Type: | Protocol |
| Date Submitted by the Author: | 17-Jul-2019 |



| | |
|---|---|
| Complete List of Authors: | ten Kate, Chantal; Erasmus University Medical Center - Sophia Children's Hospital, Paediatric Surgery<br>Vlot, John; Erasmus University Medical Center - Sophia Children's Hospital, Paediatric Surgery<br>Ijsselstijn, Hanneke ; Erasmus University Medical Center - Sophia Children's Hospital, Paediatric Surgery<br>Allegaert, Karel; Erasmus University Medical Center - Sophia Children's Hospital, Paediatrics, Division of Neonatology<br>Spaander, Manon; Erasmus University Medical Center, Gastroenterology and Hepatology<br>Poley, Marten; Erasmus University Rotterdam, Institute for Medical Technology Assessment<br>van Rosmalen, Joost; Erasmus University Medical Center, Biostatistics<br>van den Akker, Erica; Erasmus University Medical Center - Sophia Children's Hospital, Paediatric Endocrinology<br>Wijnen, Rene; Erasmus University Medical Center - Sophia Children's Hospital, Paediatric Surgery |



| | |
|---|---|
| Keywords: | oesophageal atresia, intralesional steroid injections, anastomotic strictures |


SCHOLARONE™ Manuscripts

# TITLE PAGE

**Title:**

The STEPS-EA trial: intralesional STEroid injections to Prevent refractory Strictures in patients with Esophageal Atresia – study protocol for an international, multicentre randomised controlled trial


**Authors and affiliations:**

Chantal A. ten Kate, MD[1], John Vlot, MD PhD[1], Hanneke IJsselstijn, MD PhD[1], Karel Allegaert, MD PhD[2], Manon C.W. Spaander, MD PhD[3], Marten J. Poley, PhD LLM[1, 4], Joost van Rosmalen, PhD[5], Erica L.T. van den Akker, MD PhD[6], Rene M.H. Wijnen, MD PhD[1]

[1] Department of Paediatric Surgery and Intensive Care, Erasmus MC - Sophia Children's Hospital, University Medical Centre Rotterdam, the Netherlands

[2] Department of Paediatrics, Division of Neonatology, Erasmus MC - Sophia Children's Hospital, University Medical Centre Rotterdam, the Netherlands

[3] Department of Gastroenterology and Hepatology, Erasmus MC, University Medical Centre Rotterdam, the Netherlands

[4] Institute for Medical Technology Assessment, Erasmus University Rotterdam, the Netherlands

[5] Department of Biostatistics, Erasmus MC, University Medical Centre Rotterdam, the Netherlands

[6] Department of Paediatric Endocrinology, Erasmus MC - Sophia Children's Hospital, University Medical Centre Rotterdam, the Netherlands

**Corresponding author:**

John Vlot, MD, PhD

Erasmus MC - Sophia Children's Hospital

Department of Paediatric Surgery and Intensive Care Children

Internal postal address: room Sk-1268

P.O. Box 2040, 3000 CA Rotterdam

The Netherlands

Phone: +31 10 703 62 40, Fax: +31 10 703 68 02

john.vlot@erasmusmc.nl



**Word count:** 3948 words

**Key words:** oesophageal atresia, intralesional steroid injections, anastomotic strictures




For peer review only - http://bmjopen.bmj.com/site/about/guidelines.xhtml

# ABSTRACT

**Introduction**

Anastomotic stricture formation is the most common postoperative complication after oesophageal atresia repair. The standard of care is endoscopic dilatation. A possible adjuvant treatment is intralesional steroid injection, which is thought to inhibit scar tissue formation and thereby to prevent stricture recurrence. We hypothesize that this intervention could prevent refractory strictures and reduce the total number of dilatations needed in these children.

**Methods and analysis**

This is an international multicentre randomised controlled trial. Children with oesophageal atresia type C (n=110) will be randomised into balloon dilatation followed by intralesional steroid injection or dilatation only. Randomisation and intervention will take place when a third dilatation is performed. The indication for dilatation will be confirmed with an oesophagram. One radiologist – blinded for randomisation – will review all oesophagrams. The primary outcome parameter is the total number of dilatations needed with <28 days interval, which will be analysed with a linear-by-linear chi-square association test. Secondary outcome parameters include the level of dysphagia, the luminal oesophageal diameter and stricture length as measured on the oesophagrams, the influence of co-medication on stricture formation, systemic effects of intralesional steroids (cortisol levels, length and weight) and the cost-effectiveness. Patients will undergo a second oesophagram, length and weight will be measured repeatedly, a scalp hair sample will be collected and three questionnaires will be administered. The follow-up period will be six months, with evaluation moments at 2-3 weeks, 3 months and 6 months after the intervention.

**Ethics and dissemination**

Patients will be included after written parental informed consent. The risks and burden associated with this trial are minimal. Ethical protocol has been obtained (MEC-2018-1586/NL65364.078.18). The results of the trial will be published in a peer-reviewed scientific journal as well as presented at international conferences.

**Trial registration**

EudraCT: 2018-002863-24; the Netherlands National Trial Register: NTR7726/NL7484.




For peer review only - http://bmjopen.bmj.com/site/about/guidelines.xhtml

# ARTICLE SUMMARY

**Strengths and limitations of this study**

- This is the first prospective study investigating the effectiveness of intralesional steroid injection combined with endoscopic dilatation for the treatment of recurrent esophageal strictures in children operated on for oesophageal atresia (OA).
- The determination of long term cortisol levels over a period of three months will give objective information on possible systemic effects of intralesional steroid injection in children with OA.
- Besides the effectiveness and safety of intralesional steroid injection, cost-effectiveness of this treatment will be evaluated.
- The rarity of the disease makes patient recruitment challenging.

For peer review only



For peer review only - http://bmjopen.bmj.com/site/about/guidelines.xhtml

# INTRODUCTION

Oesophageal atresia (OA) is a congenital malformation which can present with or without a tracheoesophageal fistula, with a European prevalence of 2.43 cases per 10,000 births.[1, 2] With better treatments, survival rates have increased to over 90%.[2-4] Still, anastomotic stricture formation remains the most frequent postoperative complication in up to 60% of cases.[5] Especially refractory strictures form a great burden for both patients and their parents.

The incidence of refractory strictures is poorly reported, due to the variety in definitions used in literature. Regarded as a consensus among experts, the European Society for Paediatric Gastroenterology Hepatology and Nutrition (ESPGHAN) has set the following definition for a refractory oesophageal stricture: an anatomic restriction without endoscopic inflammation that results in dysphagia after a minimum of five dilatation procedures at maximally 4-week intervals.[6, 7]

Recently, the Dutch Consortium for Esophageal Atresia (DCEA) conducted a retrospective multicentre study in the Netherlands to assess risk factors for stricture formation in children with OA. The study population consisted of 436 children born with OA between 1999 and 2013 with an end-to-end oesophageal anastomosis. Thirty-two (7.3%) of them required ≥5 dilatations within an interval of 28 days.[5]

The initial treatment of an anastomotic stricture consists of endoscopic dilatation, either balloon dilatation or semi-rigid dilatation.[6] Consensus on the preferred technique has not yet been established. A refractory stricture requires multiple dilatations under general anaesthesia, for which the child needs to be hospitalized. This adds significantly to the burden of the disease. It is therefore important to minimize the occurrence of refractory strictures and with that the need for dilatations.

In a recent ESPGHAN guideline, various adjuvant treatments, e.g. intralesional or systemic steroids, topical mitomycin C, oesophageal stents and surgical resection. The STEPS-EA trial will focus on intralesional steroid injections since we as well as the other centers involved in this trial have had successful results with this treatment in several patients.[8]

The literature on intralesional steroid injection in children with OA is scarce but promising results have been described in both children and adults with all types of oesophageal strictures (Table 1). Most studies are outdated case reports or series.[9-14] Four relatively recent randomised controlled trials (RCTs) on this topic only included adults, and with other underlying diagnoses than OA, like caustic strictures after acid ingestion, peptic strictures and anastomotic strictures after oesophagectomy with gastric tube reconstruction.[15-18] Reported beneficial effects were reduction of dilatation procedures[16], longer intervals between dilatation procedures[17], improvement of luminal diameter[15] and relief of dysphagia.[18]



For peer review only - http://bmjopen.bmj.com/site/about/guidelines.xhtml

**Table 1: Summary of literature on clinical findings on intralesional steroid injections for oesophageal strictures, including retrospective cohort studies in children <12 years (range 0-14 years).**

| Author, year | Type of study | Characteristics | Main outcomes |
|---|---|---|---|
| Camargo, 2003[15] | Double-blind RCT | 14 adult patients, corrosive strictures | • No significant difference in dilatation frequency and dysphagia.<br>• Significant improvement in obtained diameter ($p<0.05$).<br>• No adverse events reported. |
| Ramage, 2005[16] | Double-blind RCT | 30 adult patients, peptic strictures | • Less patients required repeat dilatation in the steroid group (13% vs. 60%, p=0.0209).<br>• Shorter time to repeat dilatation in control group (p=0.01).<br>• No adverse events reported. |
| Hirdes, 2013[17] | Double-blind RCT | 60 adult patients, anastomotic strictures after oesophagectomy with gastric tube reconstruction | • No significant decrease in frequency of repeat dilatation or prolongation of dysphagia-free period.<br>• Four patients developed Candida esophagitis. |
| Pereira-Lima, 2015[18] | Double-blind RCT | 19 adult patients, anastomotic strictures after oesophagectomy with gastric tube reconstruction | • Significant improvement on dysphagia at 1 and 6 months (p=0.021, p=0.009).<br>• No perforation, haemorrhage of oesophageal candidiasis. No other adverse events reported. |
| Kochar, 2002[19] | Prospective | 71 patients (13-78 year), all kinds of strictures | • Periodic dilatation index decreased significantly after injection ($p<0.001$).<br>• No adverse events reported. |
| Nijhawan, 2016[20] | Prospective | 11 adult patients, corrosive strictures | • Significant improvement of maximum dilatation ($p<0.001$) and number of dilatations per month ($p<0.001$).<br>• No adverse events reported. |
| Divarci, 2016[21] | Retrospective | 32 children (mean age 3.6 year), corrosive strictures | • Mean number of dilatation sessions was decreased (p=0.003).<br>• Mean frequency of dilatations in weeks extended ($p<0.001$).<br>• Only a positive effect in short segment strictures (<3 cm, 92% of patients dysphagia-free).<br>• No serious adverse events reported. One transient cushingoid phenotype, but no real adrenal suppression. |
| Cakmak, 2016[22] | Retrospective | 38 children (median age 1.5 year), EA (n=19) and corrosive strictures (n=19) | • No significant difference in treatment effectiveness between steroid injection and others ($p>0.05$).<br>• Intralesional steroid injections only performed in patients with long (>5 cm) and corrosive strictures & ≥5 dilatations.<br>• Four patients with oesophageal perforation, at other dilatation sessions than the intralesional steroid injection. |

For peer review only



For peer review only - http://bmjopen.bmj.com/site/about/guidelines.xhtml

In children, only retrospective studies have been performed, which mostly included caustic strictures.[21, 22] Divarci et al. analysed data of 32 children with corrosive strictures with a mean age of 3.6 years (± 2.5 years); after the intervention the number of dilatations had significantly decreased and the intervals between dilatations had extended.[21] Cakmak et al. included 38 children with either OA or corrosive strictures with a median age of 1.5 years (range 0-14 years).[22] They did not find a significant difference in treatment effectiveness between those who had received adjuvant steroid injections and the controls who had not. None of the above-mentioned studies reported any systemic effects of the local intralesional steroid injections.

All studies used triamcinolone acetonide (TAC). The exact mechanism by which TAC enhances the efficacy of dilatation is unclear. It has been proven very effective in the treatment of hypertrophic scars of the skin and keloid. A recurring anastomotic stricture can be seen as a hypertrophic lesion. The injected TAC inhibits collagen formation, enhances collagen breakdown, decreases fibrotic healing that occurs after dilatation and prevents cross-linking of collagen that causes contractions in scar tissue.[23, 24]

The recurrent dilatations and readmissions impose a substantial burden on the health care system. To date, there is no evidence on the cost-effectiveness of intralesional steroid injections. However, in the current era of evidence-based and cost-effective medicine, proof of cost-effectiveness is very relevant.

The primary objective is to evaluate whether intralesional steroid injections combined with endoscopic dilatation can prevent refractory strictures in children with OA and recurrent oesophageal stenosis, and thus can minimize the number of dilatations needed with a 28 days interval between the dilatations.

The secondary objectives are:

- to compare the level of dysphagia and the child's eating behaviour between the two groups;
- to compare the effect of intralesional steroid injections on the luminal diameter and the stricture length between the two groups;
- to evaluate a possible influence of co-medication (e.g. antacids) on stricture formation;
- to analyse the possible systemic effects of a one-time intralesional steroid injection;
- to analyse the cost-effectiveness of the use of intralesional steroid injections to prevent refractory strictures.

## METHODS AND ANALYSIS

The STEPS-EA trial is an international, multicentre, single-blinded randomised controlled trial with a 1:1 randomisation to injection with 10 mg/ml TAC (Kenacort-A 10) prior to balloon dilatation and balloon dilatation without any injection. The participating centres are tertiary (academic) hospitals that collaborate



For peer review only - http://bmjopen.bmj.com/site/about/guidelines.xhtml

within the European Reference Network on Inherited and Congenital Abnormalities (ERNICA, see also ern-ernica.eu/) and that routinely provide care for children with oesophageal atresia.

**Patient and public involvement**

Parents of patients were involved in the end-stage of the design of the trial. We presented our plans for the trial to a DCEA meeting in which also representatives of the patients association VOKS (*Vereniging voor Ouderen en Kinderen met een Slokdarmafsluiting*) take part. They were invited to comment on the study design, intervention or time required to participate in this trial. Consensus was reached on the final design during this meeting. The patients association will not be involved in the recruitment and conduct of the trial. We will involve them in dissemination, however, by presenting the trial results at a members' days or in their monthly newsletter. As the VOKS is a member of the European Federation of Esophageal Atresia and Tracheoesophageal fistula Support Groups E.V."(EAT), patients and parents throughout Europe will be informed about the results.

**Participants**

Children with OA type C[25] who underwent surgery with primary anastomosis within the first days of life in of the participating centres, and who developed a recurrent oesophageal stricture, will be eligible. Children will be included if they are ≥3 months old at the time of the intervention and are in need of a third dilatation. Written parental informed consent will be obtained by the local principal investigator (PI) or another member of the local research team. Exclusion criteria are lack of parental consent and an impossibility – known from previous dilatations – to use an endoscope with a large enough diameter working-channel to pass the endoscopic injector.

**Sample size calculation**

The power calculation is based on a linear-by-linear chi-square association test comparing the total number of dilatations required within the study period (all strictures) and within a 28-days interval (refractory strictures) between the two treatment groups. The total number of dilatations will be categorized into the categories 3, 4, 5, 6, 7-10 and >10 dilatations; a separate category for patients who are not dysphagia-free at the end of the follow-up period.

For this power calculation, we used data of the original dataset of our retrospective study in the Netherlands.[5] We selected patients from this dataset who underwent at least three dilatations within a 28 days interval (n=53). The in the retrospective study observed numbers of patients and the relative frequencies for each category are listed in Table 2. We assumed that the use of intralesional steroid injections combined with endoscopic dilatation will reduce the total number of dilatations by 50%. Note that this 50% reduction only applies to dilatations after the third dilatation, and therefore no change in the number of dilatations is assumed for the first three dilatations. This assumption leads to a different distribution for



For peer review only - http://bmjopen.bmj.com/site/about/guidelines.xhtml

the number of dilatations within the categories, which is shown as the assumed relative frequencies for the steroid group in the final column of Table 2. The details of the calculation are provided in Supplementary File 1.

In a simulation model, the required sample size to obtain a power of 80% (with a two-sided significance level of 0.05) was calculated as 52 patients per group, thus 104 in total. To account for the effects of dropout and missing data, we aim to include a total of 110 patients.

**Table 2: Assumed relative frequencies of the number of dilatations in the control and steroid groups.**

| Number of dilatations within 28 days interval | Observed number of patients (n=407)[5] | Relative frequencies control group | Assumed relative frequencies steroid group |
|---|---|---|---|
| 3 dilatations | 4 | 0.075 | 0.142 |
| 4 dilatations | 7 | 0.132 | 0.302 |
| 5 dilatations | 9 | 0.170 | 0.170 |
| 6 dilatations | 7 | 0.132 | 0.160 |
| 7-10 dilatations | 16 | 0.302 | 0.132 |
| >10 dilatations | 10 | 0.189 | 0.094 |
| Total (all numbers of dilatations combined) | 53 | 1.000 | 1.000 |

**Recruitment**

Patients will be recruited from hospitals in various European countries. Up till now, hospitals in Denmark, United Kingdom, Finland, France, Italy, Norway and Sweden have agreed to participate. During the inclusion period it will remain possible for other centres to join. To achieve adequate participant enrolment, we have minimalized the exclusion criteria. Collaboration within ERNICA should make patient accrual achievable despite the rarity of the disease.

**Randomisation, blinding and treatment allocation**

Randomisation will be conducted via ALEA (FormsVision B.V. / ALEA Clinica B.V.), a validated software program. To achieve equal distribution of the intervention among the participating sites, block randomisation stratified per centre will be carried out. The software was prepared by an independent statistician who is not otherwise involved in the study. After inclusion, the local PI will enter the patient in ALEA and will thereupon receive an email stating the allocated treatment.

Randomization will be blinded for the radiologist who will review all oesophagrams. The control group will not receive sham treatment. The steroid that will be used, Kenacort-A 10 (see below), is a white suspension, which complicates creating a placebo. Adding excipients to normal saline is undesirable, considering the unknown effect on the healing process of the stricture. Moreover, it is deemed undesirable to inject an infant with a fluid with an unknown effect.



For peer review only - http://bmjopen.bmj.com/site/about/guidelines.xhtml

**Investigational product**

The intervention is an endoscopic injection of 0.25 mL Kenacort-A 10 (Bristol-Myers Squibb BV, Utrecht, the Netherlands) in each quadrant of the stricture prior to the third endoscopic dilatation. Thus, in total, 1 mL (10 mg/ml triamcinolone acetonide, equals 12.5 mg prednisone) will be injected. During the study period, none of the patients will receive a second injection.

Kenacort-A 10 will be prepared, labelled and distributed by the trial pharmacy of the coordinating hospital. It will be delivered to the local pharmacies by courier, and the local pharmacy will deliver it to the operation room when needed. After the trial, a specific procedure for destruction of the remaining drugs is not needed; they can be disposed of locally.

**Patient timeline**

Figure 1 presents a flowchart for this study and the study procedures. The treating physician will decide on a third dilatation on the basis of the clinical signs of dysphagia and the findings on the oesophagram (thoracic x-ray with contrast, anterior-posterior and lateral). The treating physician can be a paediatric surgeon or paediatric gastroenterologist, depending on the local agreements in the different countries. After parental informed consent, the patient will be included and randomised to one of the study arms.

Prior to the balloon dilatation, an endoscopic needle (DVI-23-MH varices injector or equivalent) is pre-filled with 1 mL Kenacort-A 10 and passed through the endoscope. Under direct vision, 0.25 mL will be injected in each of the four quadrants of the circular stricture. After the injection needle has been retracted from the working channel and good visualization has been re-established, balloon dilatation will be performed up to the desired diameter. The balloon will remain insufflated for one minute.

Two to three weeks after the dilatation procedure, a second oesophagram will be made in children in both study groups. All oesophagrams will be reviewed by one specialized paediatric radiologist of the coordinating hospital, who will determine the oesophageal diameter and stricture length. Earlier studies have proven that these measurements can be obtained from an oesophagram.[26] The parents will be informed about the results and the normal standard of care will be continued.

Each patient will be followed up for six months after the third dilatation, with recording of possible side effects, complications and additional dilatations. Length and weight will be measured at 2-3 weeks, 3 months and 6 months after the third dilatation. In the context of the cost-effectiveness analysis, the parents will be asked to fill out the iMTA Productivity Cost Questionnaire (iPCQ)[27] at 3 months after the third dilatation.

At evaluation at 6 months, we will collect a scalp hair sample from the child to determine long-term cortisone and cortisol levels.[28, 29] The hair locks will be stored for final batch analysis. Last, the parents will be asked to fill out the Montreal Feeding Scale (MFS)[30-32] and again the iPCQ. After this, the study period has ended.



For peer review only - http://bmjopen.bmj.com/site/about/guidelines.xhtml

**Outcome parameters**

The primary outcome parameter is the total number of dilatations required per patient within a 28-days interval between the dilatations during the study period of six months, which is defined as the period from the day of the third dilatation until six months later.

The secondary outcome parameters are:

1) Total number of dilatations within the study period, regardless of the interval.
2) Interval (in weeks) between the start of the study and the last dilatation procedure within the study period.
3) Score on the Montreal Feeding Scale (MFS).
4) The change in maximal luminal diameter after the third dilatation relative to the diameter before the third dilatation: relative change in luminal diameter = (maximal diameter after – maximal diameter before) / maximal diameter before. The diameter will be measured at the narrowest point of the oesophagus.
5) The change in length of the oesophageal stricture after the third dilatation relative to the length before the third dilatation: relative change in stricture length = (stricture length after – stricture length before) / stricture length before. The length will be measured between the two points where the oesophageal diameter starts narrowing.
6) The use of co-medication (e.g. antacids) during the study period.
7) The mean hair cortisol levels in the first three months after the third dilatation. Cortisol levels will be adjusted for age and sex.
8) Delta length standard deviation scores (SDS) and delta weight SDS between the third dilatation (intervention) and 3 and 6 months after the third dilatation.
9) Total costs of the treatment, including medical and non-medical costs.
10) Incremental costs per refractory stricture prevented and incremental costs per additional dysphagia-free patient.

For peer review only

**Data collection**

All participating centres are familiar with the procedure of injecting Kenacort-A 10 in the lesion via an injection needle through the endoscope. However, to guarantee equality of the intervention, the relevant practitioner(s) in all centres will be trained by the PI of the trial. Radiologic interobserver variability will be avoided by having all oesophagrams reviewed by one radiologist.

The internationally validated MFS[30-32] will be used to measure dysphagia. The MFS has often been used in previous research in children with OA. The scalp hair sample will be taken from the posterior vertex; cortisol levels will be determined with the liquid chromatography-tandem mass spectrometry (LC-MS/MS) method for quantification of steroids.[28]



For peer review only - http://bmjopen.bmj.com/site/about/guidelines.xhtml

The cost-effectiveness analysis (CEA) will follow established methods for economic evaluations and costing studies in health care.[33, 34] Both medical and non-medical costs will be collected. Medical costs will include costs of surgeries (dilatations), steroid injections, hospital days (on the ward or the intensive care unit), medication (such as analgesics) and diagnostic radiography. Costs of health care provided by others than the participating centres (such as other hospitals or general practitioners) will be ignored in this study, as these are unlikely to be affected by the intervention. Non-medical costs will include costs of special diets, costs related to hospital visits (i.e. costs of transportation and costs of paid childminders for siblings), and productivity losses related to both paid and unpaid work. The non-medical costs will be measured using the iPCQ[27], supplemented with additional questions on costs of special diets and costs related to the child's hospitalisation. The original iPCQ questions are validated in English. The complete questionnaire including the additional questions will be translated to the languages required for this trial using the forward-backward translation method, as will the MFS for the languages it has not been validated for.

**Statistical analysis**

Since a standardized treatment protocol will be adopted in all centres, statistical adjustment for centre effects is considered unnecessary.

The primary outcome parameter will be analysed with a linear-by-linear chi-square association test. The total number of dilatations required within a 28 days interval (i.e. refractory strictures) within the study period of 6 months per patient will be categorized and compared between treatment groups. In case of death during the follow-up period, the outcome will be set to the highest (i.e. most severe) category. In case of drop-out during the follow-up period due to other causes (e.g. emigration, withdrawal), the subject will be excluded from the study.

The analyses for the secondary study parameters are as follows:

1) The total number of dilatations required within the study period (regardless of the interval, i.e. all strictures) will be categorized and compared between treatment groups with a linear-by-linear chi-square association test.
2) The interval (in weeks) until the patient is dysphagia-free will be compared between groups with the log-rank test and with Cox proportional hazards regression, with adjustment for treatment group and factors such as age, sex, diagnostic information and described risk factors for stricture formation like anastomotic leaking and thoracoscopic repair.[5] Patients who do not become dysphagia-free during follow-up are treated as censored at the end of the follow-up period.
3) The scores on the MFS (reflecting the level of dysphagia and the eating behaviour) will be compared between study groups with a Mann-Whitney test.
4) The relative change in the oesophageal diameter and that in the length of the oesophageal stricture will be compared between study groups with an analysis of covariance model. The dependent variables in this model will be the log-transformed oesophageal diameter and length after third



For peer review only - http://bmjopen.bmj.com/site/about/guidelines.xhtml

dilatation, and the independent variables the treatment group and the log-transformed oesophageal diameter and length before third dilatation.

6) The effect of co-medication on the primary study outcome will be assessed using a stratified Mann-Whitney test with stratification for the treatment group.
7) The mean cortisol level over the first three months after the third dilatation will be compared between study groups with a linear regression model with adjustment for age and sex.

No missing data are expected for the independent variables in the analysis of covariance models and the Cox proportional hazards regression models. In case of missing data for any of the outcomes, a complete case analysis (i.e. exclusion of the subjects who dropped out during the follow-up period) will be performed for the corresponding outcome.

**Cost-effectiveness analysis**

All medical and non-medical costs will be summated for each individual patient. Regarding the patient outcomes, the number of refractory strictures prevented and the number of dysphagia-free patients will be considered. Incremental cost-effectiveness ratios will be calculated, expressed as incremental costs per refractory stricture prevented and incremental costs per additional dysphagia-free patient. Analysis of uncertainty will be illustrated through cost-effectiveness planes (via bootstrapping).

As this RCT is done in an international setting, it must be recognized that the results may differ between countries because health care systems, treatment patterns and prices may vary. Therefore, country-level information will be collected. Data (especially resource quantities and cost prices) will be collected in all countries, and results will also be reported for each country separately. Next, a pooled summary calculation of the intervention's cost-effectiveness will be made, converting all costs into a common currency base (i.e. Euros).

# ETHICS AND DISSEMINATION

**Ethical consideration**

This study protocol was approved by the Institutional Review Board (IRB) of the Erasmus Medical Centre (MEC-2018-1586/NL65364.078.18). In case of any modifications of the protocol, a formal amendment will be submitted to the IRB. Approved changes will be communicated to all relevant parties according to the rules of the IRB. The protocol has currently been submitted for local ethical approval in Amsterdam (the Netherlands), Helsinki (Finland), Odense (Denmark), Copenhagen (Denmark), Stockholm (Sweden) and Padua (Italy). Nijmegen (the Netherlands) has already obtained local ethical approval and joined the study. To guarantee the respect of ethical rules and standard of care in all participating centres, the protocol was reviewed by the two chairs of the work package on oesophageal atresia within ERNICA. The informed consent and assent process of this trial is in line with the Good Clinical Practice guideline.[35]



For peer review only - http://bmjopen.bmj.com/site/about/guidelines.xhtml

For peer review only

Furthermore, we involved external experts in the form of a Data Safety Monitoring Board (DSMB). These experts are a paediatric surgeon, a pharmacologist and a statistician. All experts are independent of the sponsor and therefore competing interests will be avoided. The DSMB will monitor the safety of the study subjects and data. The board will meet at least three times: within one year of recruitment commencing, at the time of the planned interim analyses at 50% (n=55) of enrolment and at the conclusion of the trial.

**Adverse events and auditing**

Adverse events will be handled according to the guidelines of the IRB of the Erasmus Medical Centre. All adverse events will be registered during the study. Serious adverse events will be reported to the sponsor immediately and registered appropriately within 24 hours.

All participating sites will be audited once a year with monitoring of patient recruitment, source data verification, drug accountability and sample storage. The auditor will be independent and not involved in the study.

**Benefits and risk assessment**

The risks and burden associated with this study are minimal. Potential complications of oesophageal steroid injections include adrenal suppression, perforation, intramural infection, candida infection, mediastinitis and pleural effusion.[36] However, in previous studies no adverse events have been reported in relation to the steroid injections (see Table 1). Additionally, Kenacort-A 10 is a depot preparation with a slow release and therefore a gradual exposure. This implies that the likelihood of acute exposure to a high dose of steroids will be minimal.

The burden of filling out the questionnaires and taking a hair sample are negligible. Filling out the MFS and iPCQ will take maximally 30 minutes. All participants will undergo one extra oesophagram after the third dilatation procedure. The potential reduction in number of anaesthetic procedures needed for dilatations outweighs the burden and the radiation exposure of this oesophagram. Potential benefits of intralesional steroid injections in anastomotic strictures in children with OA are fewer dilatation procedures needed, with concomitant fewer anaesthetic procedures and hospital admissions, and less risk for perforation.

**Data management**

All data will be handled confidentially and anonymously using OpenClinica V3.12.2 (OpenClinica LLC, USA) for data collection. The questionnaires will be conducted through an online survey using LimeSurvey (LimeSurvey GmbH, Germany) and GemsTracker (Erasmus MC, Rotterdam, the Netherlands). All patient data will be coded using a subject identification code list. The local PI safeguards the key to the code; the sponsor will have access to these codes. The local PI will only have access to the data of patients of their own centre; the sponsor will have access to the final trial dataset. All has been stated in a clinical trial site agreement, signed by all participating sites.



For peer review only - http://bmjopen.bmj.com/site/about/guidelines.xhtml

**Dissemination**

The results of this trial will be published in an international peer-reviewed scientific journal, within one year after the end of the follow-up period of the last included patient. In addition, we aim to present the results at several international conferences, to inform health care professionals worldwide.

## TRIAL STATUS

The study has started in Rotterdam (the Netherlands) in February 2019, after ethical approval had been obtained. Nijmegen (the Netherlands) has joined the study in the summer of 2019. The first patient has yet to be included. At this moment, Amsterdam (the Netherlands), Helsinki (Finland), Odense (Denmark), Copenhagen (Denmark), Stockholm (Sweden) and Padua (Italy) are still waiting for local ethical approval. We expect to start the study in these sites the latest at the end of 2019.

Considering the rarity of the study, we expect to complete the inclusions and finish data collection for this study in five years.

For peer review only



For peer review only - http://bmjopen.bmj.com/site/about/guidelines.xhtml

## ACKNOWLEDGEMENTS

The author thank Benno Ure, paediatric surgeon and head of department of Paediatric Surgery of Hannover Medical School, and Frédéric Gottrand, paediatrician at the department of Paediatric Gastroenterology of University Hospital of Lille, for their input during the development of the trial protocol. The authors thank Joke Dunk, research nurse at the Erasmus University Medical Centre in Rotterdam, for her help with the implementation of the trial. Ko Hagoort provided editorial advice. At last, we thank all hospitals involved in ERNICA and the VOKS for their input and collaboration.

## AUTHORS CONTRIBUTIONS

RW is the initiator of the study. CK (PhD candidate), JV (paediatric surgeon), HIJ (paediatrician), KA (paediatric clinical pharmacologist), MS (gastroenterologist), MP (health economist), JR (statistician), EA (paediatric endocrinologist) and RW (paediatric surgeon) contributed in writing and designing of the study protocol. JV (principal investigator) and RW (project leader) will supervise the study. CK will coordinate the study and be responsible for data acquisition. CK, JV and JR will carry out the analyses. All authors have read and approved the final version of the study protocol and manuscript.

## FUNDING STATEMENT

This research received no specific grant from any funding agency in the public, commercial or not-for-profit sectors.

## COMPETING INTEREST STATEMENT

None declared.

## LICENSE STATEMENT

I, the Submitting Author has the right to grant and does grant on behalf of all authors of the Work (as defined in the below author licence), an exclusive licence and/or a non-exclusive licence for contributions from authors who are: i) UK Crown employees; ii) where BMJ has agreed a CC-BY licence shall apply, and/or iii) in accordance with the terms applicable for US Federal Government officers or employees acting as part of their official duties; on a worldwide, perpetual, irrevocable, royalty-free basis to BMJ Publishing Group Ltd ("BMJ") its licensees and where the relevant Journal is co-owned by BMJ to the co-owners of the Journal, to publish the Work in BMJ Open and any other BMJ products and to exploit all rights, as set out in our licence.

The Submitting Author accepts and understands that any supply made under these terms is made by BMJ to the Submitting Author unless you are acting as an employee on behalf of your employer or a postgraduate



For peer review only - http://bmjopen.bmj.com/site/about/guidelines.xhtml

student of an affiliated institution which is paying any applicable article publishing charge ("APC") for Open Access articles. Where the Submitting Author wishes to make the Work available on an Open Access basis (and intends to pay the relevant APC), the terms of reuse of such Open Access shall be governed by a Creative Commons licence – details of these licences and which Creative Commons licence will apply to this Work are set out in our licence referred to above.

## DATA AVAILABILIY STATEMENT

The data that support the findings of this trial will be available from the corresponding author upon reasonable request once the results are published.

For peer review only

# REFERENCES


1. Pedersen RN, Markow S, Kruse-Andersen S, et al. Esophageal atresia: gastroesophageal functional follow-up in 5-15 year old children. *Journal of Pediatric Surgery* 2013;48(12):2487-95.
2. Oddsberg J, Lu Y, Lagergren J. Aspects of esophageal atresia in a population-based setting: incidence, mortality, and cancer risk. *Pediatr Surg Int* 2012;28(3):249-57.
3. Wang B, Tashiro J, Allan BJ, et al. A nationwide analysis of clinical outcomes among newborns with esophageal atresia and tracheoesophageal fistulas in the United States. *J Surg Res* 2014;190(2):604-12.
4. Sulkowski JP, Cooper JN, Lopez JJ, et al. Morbidity and mortality in patients with esophageal atresia. *Surgery* 2014;156(2):483-91.
5. Vergouwe FWT, Vlot J, H IJ, et al. Risk factors for refractory anastomotic strictures after oesophageal atresia repair: a multicentre study. *Arch Dis Child* 2018.
6. Krishnan U, Mousa H, Dall'Oglio L, et al. ESPGHAN-NASPGHAN Guidelines for the Evaluation and Treatment of Gastrointestinal and Nutritional Complications in Children With Esophageal Atresia-Tracheoesophageal Fistula. *J Pediatr Gastroenterol Nutr* 2016;63(5):550-70.
7. Tringali A, Thomson M, Dumonceau JM, et al. Pediatric gastrointestinal endoscopy: European Society of Gastrointestinal Endoscopy (ESGE) and European Society for Paediatric Gastroenterology Hepatology and Nutrition (ESPGHAN) Guideline Executive summary. *Endoscopy* 2017;49(1):83-91.
8. Ten Kate CA, Vlot J, Sloots CEJ, et al. The effect of intralesional steroid injections on esophageal strictures and the child as whole: a case series. *J Pediatr Surg* 2019.
9. Holder TM, Ashcraft KW, Leape L. The treatment of patients with esophageal strictures by local steroid injections. *J Pediatr Surg* 1969;4(6):646-53.
10. Zein NN, Greseth JM, Perrault J. Endoscopic intralesional steroid injections in the management of refractory esophageal strictures. *Gastrointest Endosc* 1995;41(6):596-8.
11. Mendelsohn HJ, Maloney WH. The treatment of benign strictures of the esophagus with cortisone injection. *Ann Otol Rhinol Laryngol* 1970;79(5):900-4.
12. Gandhi RP, Cooper A, Barlow BA. Successful management of esophageal strictures without resection or replacement. *J Pediatr Surg* 1989;24(8):745-9; discussion 49-50.
13. Kirsch M, Blue M, Desai RK, et al. Intralesional steroid injections for peptic esophageal strictures. *Gastrointest Endosc* 1991;37(2):180-2.
14. Kochhar R, Ray JD, Sriram PV, et al. Intralesional steroids augment the effects of endoscopic dilation in corrosive esophageal strictures. *Gastrointest Endosc* 1999;49(4 Pt 1):509-13.
15. Camargo MA, Lopes LR, Grangeia Tde A, et al. [Use of corticosteroids after esophageal dilations on patients with corrosive stenosis: prospective, randomized and double-blind study]




For peer review only - http://bmjopen.bmj.com/site/about/guidelines.xhtml

O uso de corticoesteroides apos dilata o esofagica em pacientes portadores de estenose por substancias corrosivas: estudo prospectivo, randomizado e duplo-cego. *Rev Assoc Med Bras (1992)* 2003;49(3):286-92.

16. Ramage JI, Jr., Rumalla A, Baron TH, et al. A prospective, randomized, double-blind, placebo-controlled trial of endoscopic steroid injection therapy for recalcitrant esophageal peptic strictures. *Am J Gastroenterol* 2005;100(11):2419-25.
17. Hirdes MM, van Hooft JE, Koornstra JJ, et al. Endoscopic corticosteroid injections do not reduce dysphagia after endoscopic dilation therapy in patients with benign esophagogastric anastomotic strictures. *Clin Gastroenterol Hepatol* 2013;11(7):795-801 e1.
18. Pereira-Lima JC, Lemos Bonotto M, Hahn GD, et al. A prospective randomized trial of intralesional triamcinolone injections after endoscopic dilation for complex esophagogastric anastomotic strictures: steroid injection after endoscopic dilation. *Surg Endosc* 2015;29(5):1156-60.
19. Kochhar R, Makharia GK. Usefulness of intralesional triamcinolone in treatment of benign esophageal strictures. *Gastrointest Endosc* 2002;56(6):829-34.
20. Nijhawan S, Udawat HP, Nagar P. Aggressive bougie dilatation and intralesional steroids is effective in refractory benign esophageal strictures secondary to corrosive ingestion. *Dis Esophagus* 2016;29(8):1027-31.
21. Divarci E, Celtik U, Dokumcu Z, et al. The Efficacy of Intralesional Steroid Injection in the Treatment of Corrosive Esophageal Strictures in Children. *Surg Laparosc Endosc Percutan Tech* 2016;26(6):e122-e25.
22. Cakmak M, Boybeyi O, Gollu G, et al. Endoscopic balloon dilatation of benign esophageal strictures in childhood: a 15-year experience. *Dis Esophagus* 2016;29(2):179-84.
23. Ashcraft KW, Holder TM. The expeimental treatment of esophageal strictures by intralesional steroid injections. *J Thorac Cardiovasc Surg* 1969;58(5):685-91 passim.
24. Ketchum LD, Smith J, Robinson DW, et al. The treatment of hypertrophic scar, keloid and scar contracture by triamcinolone acetonide. *Plast Reconstr Surg* 1966;38(3):209-18.
25. Gross RE. Atresia of the esophagus. *Am J Dis Child* 1947;74(3):369.
26. Sun LY, Laberge JM, Yousef Y, et al. The Esophageal Anastomotic Stricture Index (EASI) for the management of esophageal atresia. *J Pediatr Surg* 2015;50(1):107-10.
27. Bouwmans C, Krol M, Severens H, et al. The iMTA Productivity Cost Questionnaire: A Standardized Instrument for Measuring and Valuing Health-Related Productivity Losses. *Value Health* 2015;18(6):753-8.
28. Noppe G, de Rijke YB, Dorst K, et al. LC-MS/MS-based method for long-term steroid profiling in human scalp hair. *Clin Endocrinol (Oxf)* 2015;83(2):162-6.
29. Wester VL, Noppe G, Savas M, et al. Hair analysis reveals subtle HPA axis suppression associated with use of local corticosteroids: The Lifelines cohort study. *Psychoneuroendocrinology* 2017;80:1-6.



For peer review only - http://bmjopen.bmj.com/site/about/guidelines.xhtml

30. Baird R, Levesque D, Birnbaum R, et al. A pilot investigation of feeding problems in children with esophageal atresia. *Dis Esophagus* 2015;28(3):224-8.
31. Ramsay M, Martel C, Porporino M, et al. The Montreal Children's Hospital Feeding Scale: A brief bilingual screening tool for identifying feeding problems. *Paediatr Child Health* 2011;16(3):147-e17.
32. Van Dijk M, Timmerman M, Martel C, et al. Towards the development of a Dutch screening instrument for the detection of feeding problems in young children. *Netherlands Journal of Psychology* 2011;66:112-19.
33. National Health Institute Diemen. Guidelines for economic evaluations of health care [in Dutch].
34. National Health Care Institute Diemen. Manual for costing research. Methods and reference prices for economic evaluations in health care. 2015 Updated edition [in Dutch]. .
35. ICH Expert Working Group. Guideline for Good Clinical Practice. 1996.
36. Kochhar R, Poornachandra KS. Intralesional steroid injection therapy in the management of resistant gastrointestinal strictures. *World J Gastrointest Endosc* 2010;2(2):61-8.

For peer review only



For peer review only - http://bmjopen.bmj.com/site/about/guidelines.xhtml

# FIGURE LEGENDS

**Figure 1: Flowchart of the study design**

OA = oesophageal atresia, AP = anterior-posterior, iPCQ = iMTA Productivity Cost Questionnaire, MFS = Montreal Feeding Scale. **Bold with underline** = study procedures, the rest is standard of care.

For peer review only

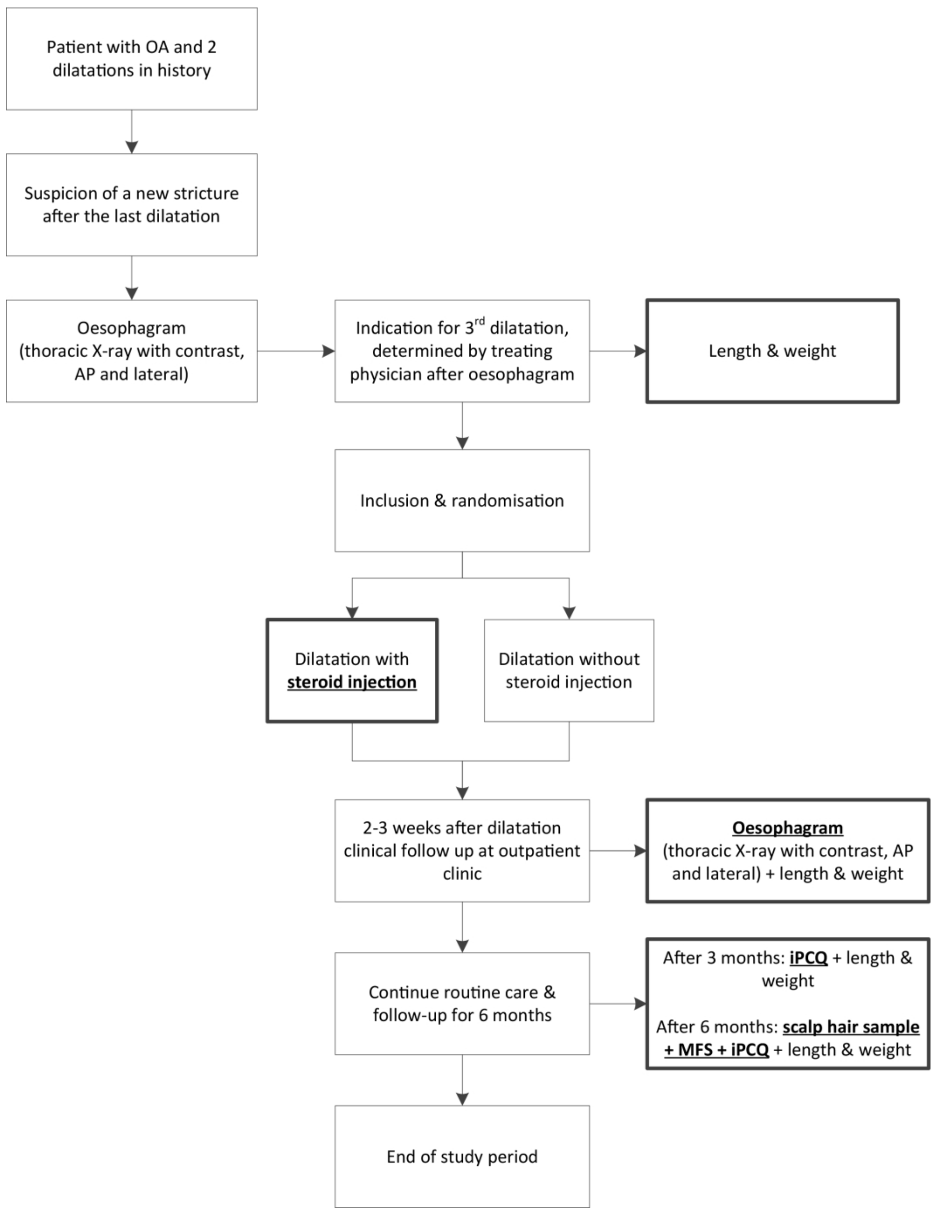


Figure 1: Flowchart of the study design
OA = oesophageal atresia, AP = anterior-posterior, iPCQ = iMTA Productivity Cost Questionnaire, MFS = Montreal Feeding Scale. Bold with underline = study procedures, the rest is standard of care.

# SUPPLEMENTARY FILE

To calculate the distribution of the number of dilatations in the steroid group, we assumed the following:

- The first 3 dilatations are unaffected by the intervention; thus the 4 patients with 3 dilatations in the observed data would still have 3 dilatations with steroid treatment.
- The 7 patients with 4 dilatations in the observed data would, if steroid treatment were applied, be equally divided over the categories 3 dilatations and 4 dilatations (3.5 patients in each category).
- The 9 patients with 5 dilatations in the observed data would, if steroid treatment were applied, have 4 dilatations.
- The 7 patients with 6 dilatations in the observed data would, if steroid treatment were applied, be equally divided over the categories 4 dilatations and 5 dilatations (3.5 patients in each category).
- The 16 patients with 7-10 dilatations in the observed data would, if steroid treatment were applied, be divided over the categories 5, 6 and 7-10 dilatations (patients with 7 dilatations would have 5 dilatations, patients with 8 dilatations would have 5 or 6 dilatations, patients with 9 dilatations would have 6 dilatations and patients with 10 dilatations would have 6 or 7 dilatations).
- The 10 patients with >10 dilatations in the observed data would, if steroid treatment were applied, be divided over the categories 7-10 and >10 dilatations (5 patients in each category).

These assumptions lead to the predicted numbers of patients shown in Supplementary Table 1. The relative frequency distribution in the steroid group is then calculated by dividing the predicted numbers of patients by the total number of 53 patients.

| Number of dilatations within 28 days interval | Observed number of patients (n=407) [1] | Predicted number of patients with steroid treatment | Relative frequencies control group | Assumed relative frequencies steroid group |
|---|---|---|---|---|
| 3 dilatations | 4 | 7.5 (4+3.5) | 0.075 | 0.142 |
| 4 dilatations | 7 | 16 (3.5+9+3.5) | 0.132 | 0.302 |
| 5 dilatations | 9 | 9 (3.5+2+3.5) | 0.170 | 0.170 |
| 6 dilatations | 7 | 8.5 (3.5+3+2) | 0.132 | 0.160 |
| 7-10 dilatations* | 16 | 7 (2+5) | 0.302 | 0.132 |
| >10 dilatations* | 10 | 5 (5) | 0.189 | 0.094 |
| Total (all numbers of dilatations combined) | 53 | 53 | 1.000 | 1.000 |

**Supplementary Table 1:** Assumed relative frequencies of the number of dilatations in the control and steroid groups, including the calculated predicted number of patients with steroid treatment. *See Supplementary Table 2 for the exact number of dilatations within these categories.

For peer review only

| **Number of dilatations within 28 days interval** | **Observed number of patients (n=407)[1]** |
|---|---|
| 3 dilatations | 4 |
| 4 dilatations | 7 |
| 5 dilatations | 9 |
| 6 dilatations | 7 |
| 7 dilatations | 2 |
| 8 dilatations | 7 |
| 9 dilatations | 3 |
| 10 dilatations | 4 |
| 12 dilatations | 2 |
| 13 dilatations | 2 |
| 15 dilatations | 1 |
| 18 dilatations | 2 |
| 24 dilatations | 1 |
| 30 dilatations | 1 |
| 34 dilatations | 1 |
| Total (all numbers of dilatations combined) | 53 |

**Supplementary Table 2:** *Exact* number of dilatations as extracted from the *original dataset* of the retrospective study in the Netherlands.[1]

1. Vergouwe FWT, Vlot J, H IJ, et al. Risk factors for refractory anastomotic strictures after oesophageal atresia repair: a multicentre study. *Arch Dis Child* 2018.

For peer review only

# Reporting checklist for protocol of a clinical trial.

Based on the SPIRIT guidelines.

## Instructions to authors

Complete this checklist by entering the page numbers from your manuscript where readers will find each of the items listed below.

Your article may not currently address all the items on the checklist. Please modify your text to include the missing information. If you are certain that an item does not apply, please write "n/a" and provide a short explanation.

Upload your completed checklist as an extra file when you submit to a journal.

In your methods section, say that you used the SPIRITreporting guidelines, and cite them as:

Chan A-W, Tetzlaff JM, Altman DG, Laupacis A, Gøtzsche PC, Krleža-Jerić K, Hróbjartsson A, Mann H, Dickersin K, Berlin J, Doré C, Parulekar W, Summerskill W, Groves T, Schulz K, Sox H, Rockhold FW, Rennie D, Moher D. SPIRIT 2013 Statement: Defining standard protocol items for clinical trials. Ann Intern Med. 2013;158(3):200-207

| | | Reporting Item | Page Number |
|---|---|---|---|
| **Administrative information** | | | |
| Title | #1 | Descriptive title identifying the study design, population, interventions, and, if applicable, trial acronym | 1 |
| Trial registration | #2a | Trial identifier and registry name. If not yet registered, name of intended registry | 2 |
| Trial registration: data set | #2b | All items from the World Health Organization Trial Registration Data Set | 2 |
| Protocol version | #3 | Date and version identifier | 2 |
| Funding | #4 | Sources and types of financial, material, and other support | 15 |
| Roles and responsibilities: contributorship | #5a | Names, affiliations, and roles of protocol contributors | 15 |

For peer review only

| | | | |
|---|---|---|---|
| Roles and responsibilities: sponsor contact information | #5b | Name and contact information for the trial sponsor | 1 |
| Roles and responsibilities: sponsor and funder | #5c | Role of study sponsor and funders, if any, in study design; collection, management, analysis, and interpretation of data; writing of the report; and the decision to submit the report for publication, including whether they will have ultimate authority over any of these activities | 15 |
| Roles and responsibilities: committees | #5d | Composition, roles, and responsibilities of the coordinating centre, steering committee, endpoint adjudication committee, data management team, and other individuals or groups overseeing the trial, if applicable (see Item 21a for data monitoring committee) | 15 |
| **Introduction** | | | |
| Background and rationale | #6a | Description of research question and justification for undertaking the trial, including summary of relevant studies (published and unpublished) examining benefits and harms for each intervention | 4-6 |
| Background and rationale: choice of comparators | #6b | Explanation for choice of comparators | 4-6 |
| Objectives | #7 | Specific objectives or hypotheses | 6 |
| Trial design | #8 | Description of trial design including type of trial (eg, parallel group, crossover, factorial, single group), allocation ratio, and framework (eg, superiority, equivalence, non-inferiority, exploratory) | 6-7 |
| **Methods: Participants, interventions, and outcomes** | | | |
| Study setting | #9 | Description of study settings (eg, community clinic, academic hospital) and list of countries where data will be collected. Reference to where list of study sites can be obtained | 6-7 |
| Eligibility criteria | #10 | Inclusion and exclusion criteria for participants. If applicable, eligibility criteria for study centres and individuals who will | 7 |

For peer review only

| | | | |
|---|---|---|---|
| | | perform the interventions (eg, surgeons, psychotherapists) | |
| Interventions: description | #11a | Interventions for each group with sufficient detail to allow replication, including how and when they will be administered | 9 |
| Interventions: modifications | #11b | Criteria for discontinuing or modifying allocated interventions for a given trial participant (eg, drug dose change in response to harms, participant request, or improving / worsening disease) | N/A |
| Interventions: adherance | #11c | Strategies to improve adherence to intervention protocols, and any procedures for monitoring adherence (eg, drug tablet return; laboratory tests) | N/A |
| Interventions: concomitant care | #11d | Relevant concomitant care and interventions that are permitted or prohibited during the trial | 9 |
| Outcomes | #12 | Primary, secondary, and other outcomes, including the specific measurement variable (eg, systolic blood pressure), analysis metric (eg, change from baseline, final value, time to event), method of aggregation (eg, median, proportion), and time point for each outcome. Explanation of the clinical relevance of chosen efficacy and harm outcomes is strongly recommended | 10 |
| Participant timeline | #13 | Time schedule of enrolment, interventions (including any run-ins and washouts), assessments, and visits for participants. A schematic diagram is highly recommended (see Figure) | 9 |
| Sample size | #14 | Estimated number of participants needed to achieve study objectives and how it was determined, including clinical and statistical assumptions supporting any sample size calculations | 7-8 |
| Recruitment | #15 | Strategies for achieving adequate participant enrolment to reach target sample size | 8 |
| **Methods: Assignment of interventions (for controlled trials)** | | | |
| Allocation: sequence generation | #16a | Method of generating the allocation sequence (eg, computer-generated random numbers), and list of any factors for stratification. To reduce predictability of a random sequence, details of any planned restriction (eg, blocking) should be provided in a separate document that is unavailable to those who enrol participants or assign interventions | 8 |

For peer review only

| | | | |
|---|---|---|---|
| Allocation concealment mechanism | #16b | Mechanism of implementing the allocation sequence (eg, central telephone; sequentially numbered, opaque, sealed envelopes), describing any steps to conceal the sequence until interventions are assigned | 8 |
| Allocation: implementation | #16c | Who will generate the allocation sequence, who will enrol participants, and who will assign participants to interventions | 8 |
| Blinding (masking) | #17a | Who will be blinded after assignment to interventions (eg, trial participants, care providers, outcome assessors, data analysts), and how | 8 |
| Blinding (masking): emergency unblinding | #17b | If blinded, circumstances under which unblinding is permissible, and procedure for revealing a participant's allocated intervention during the trial | N/A |
| **Methods: Data collection, management, and analysis** | | | |
| Data collection plan | #18a | Plans for assessment and collection of outcome, baseline, and other trial data, including any related processes to promote data quality (eg, duplicate measurements, training of assessors) and a description of study instruments (eg, questionnaires, laboratory tests) along with their reliability and validity, if known. Reference to where data collection forms can be found, if not in the protocol | 10-11 |
| Data collection plan: retention | #18b | Plans to promote participant retention and complete follow-up, including list of any outcome data to be collected for participants who discontinue or deviate from intervention protocols | 10-11 |
| Data management | #19 | Plans for data entry, coding, security, and storage, including any related processes to promote data quality (eg, double data entry; range checks for data values). Reference to where details of data management procedures can be found, if not in the protocol | 10-11 |
| Statistics: outcomes | #20a | Statistical methods for analysing primary and secondary outcomes. Reference to where other details of the statistical analysis plan can be found, if not in the protocol | 11-12 |
| Statistics: additional analyses | #20b | Methods for any additional analyses (eg, subgroup and adjusted analyses) | 11-12 |

For peer review only

| | | | |
|---|---|---|---|
| Statistics: analysis population and missing data | #20c | Definition of analysis population relating to protocol non-adherence (eg, as randomised analysis), and any statistical methods to handle missing data (eg, multiple imputation) | 11-12 |
| **Methods: Monitoring** | | | |
| Data monitoring: formal committee | #21a | Composition of data monitoring committee (DMC); summary of its role and reporting structure; statement of whether it is independent from the sponsor and competing interests; and reference to where further details about its charter can be found, if not in the protocol. Alternatively, an explanation of why a DMC is not needed | 13 |
| Data monitoring: interim analysis | #21b | Description of any interim analyses and stopping guidelines, including who will have access to these interim results and make the final decision to terminate the trial | 13 |
| Harms | #22 | Plans for collecting, assessing, reporting, and managing solicited and spontaneously reported adverse events and other unintended effects of trial interventions or trial conduct | 13 |
| Auditing | #23 | Frequency and procedures for auditing trial conduct, if any, and whether the process will be independent from investigators and the sponsor | 13 |
| **Ethics and dissemination** | | | |
| Research ethics approval | #24 | Plans for seeking research ethics committee / institutional review board (REC / IRB) approval | 12 |
| Protocol amendments | #25 | Plans for communicating important protocol modifications (eg, changes to eligibility criteria, outcomes, analyses) to relevant parties (eg, investigators, REC / IRBs, trial participants, trial registries, journals, regulators) | 12 |
| Consent or assent | #26a | Who will obtain informed consent or assent from potential trial participants or authorised surrogates, and how (see Item 32) | 7 |
| Consent or assent: ancillary studies | #26b | Additional consent provisions for collection and use of participant data and biological specimens in ancillary studies, if applicable | N/A |
| Confidentiality | #27 | How personal information about potential and enrolled participants will be collected, shared, and maintained in order to protect confidentiality before, during, and after the trial | 10-11 |

For peer review only

| | | | |
|---|---|---|---|
| Declaration of interests | #28 | Financial and other competing interests for principal investigators for the overall trial and each study site | 15 |
| Data access | #29 | Statement of who will have access to the final trial dataset, and disclosure of contractual agreements that limit such access for investigators | 10-11 |
| Ancillary and post trial care | #30 | Provisions, if any, for ancillary and post-trial care, and for compensation to those who suffer harm from trial participation | N/A |
| Dissemination policy: trial results | #31a | Plans for investigators and sponsor to communicate trial results to participants, healthcare professionals, the public, and other relevant groups (eg, via publication, reporting in results databases, or other data sharing arrangements), including any publication restrictions | 14 |
| Dissemination policy: authorship | #31b | Authorship eligibility guidelines and any intended use of professional writers | 14 |
| Dissemination policy: reproducible research | #31c | Plans, if any, for granting public access to the full protocol, participant-level dataset, and statistical code | 14 |
| **Appendices** | | | |
| Informed consent materials | #32 | Model consent form and other related documentation given to participants and authorised surrogates | N/A |
| Biological specimens | #33 | Plans for collection, laboratory evaluation, and storage of biological specimens for genetic or molecular analysis in the current trial and for future use in ancillary studies, if applicable | N/A |

The SPIRIT checklist is distributed under the terms of the Creative Commons Attribution License CC-BY-ND 3.0. This checklist was completed on 15. July 2019 using https://www.goodreports.org/, a tool made by the EQUATOR Network in collaboration with Penelope.ai

For peer review only

BMJ Open

# The STEPS-EA trial: intralesional STEroid injections to Prevent refractory Strictures in patients with Esophageal Atresia – study protocol for an international, multicentre randomised controlled trial



| | |
|---|---|
| Journal: | *BMJ Open* |
| Manuscript ID | bmjopen-2019-033030.R1 |
| Article Type: | Protocol |
| Date Submitted by the Author: | 06-Nov-2019 |



| | |
|---|---|
| Complete List of Authors: | ten Kate, Chantal; Erasmus University Medical Center - Sophia Children's Hospital, Paediatric Surgery<br>Vlot, John; Erasmus University Medical Center - Sophia Children's Hospital, Paediatric Surgery<br>Ijsselstijn, Hanneke ; Erasmus University Medical Center - Sophia Children's Hospital, Paediatric Surgery<br>Allegaert, Karel; Erasmus University Medical Center - Sophia Children's Hospital, Paediatrics, Division of Neonatology<br>Spaander, Manon; Erasmus University Medical Center, Gastroenterology and Hepatology<br>Poley, Marten; Erasmus University Rotterdam, Institute for Medical Technology Assessment<br>van Rosmalen, Joost; Erasmus University Medical Center, Biostatistics<br>van den Akker, Erica; Erasmus University Medical Center - Sophia Children's Hospital, Paediatric Endocrinology<br>Wijnen, Rene; Erasmus University Medical Center - Sophia Children's Hospital, Paediatric Surgery |



| | |
|---|---|
| <b>Primary Subject Heading</b>: | Surgery |
| Secondary Subject Heading: | Paediatrics |
| Keywords: | oesophageal atresia, intralesional steroid injections, anastomotic strictures |
| | |


SCHOLARONE™ Manuscripts

# TITLE PAGE

**Title:**

The STEPS-EA trial: intralesional STEroid injections to Prevent refractory Strictures in patients with Esophageal Atresia – study protocol for an international, multicentre randomised controlled trial

**Authors and affiliations:**

Chantal A. ten Kate, MD[1], John Vlot, MD PhD[1], Hanneke IJsselstijn, MD PhD[1], Karel Allegaert, MD PhD[2], Manon C.W. Spaander, MD PhD[3], Marten J. Poley, PhD LLM[1, 4], Joost van Rosmalen, PhD[5], Erica L.T. van den Akker, MD PhD[6], Rene M.H. Wijnen, MD PhD[1]

[1] Department of Paediatric Surgery and Intensive Care, Erasmus MC - Sophia Children's Hospital, University Medical Centre Rotterdam, the Netherlands

[2] Department of Paediatrics, Division of Neonatology, Erasmus MC - Sophia Children's Hospital, University Medical Centre Rotterdam, the Netherlands

[3] Department of Gastroenterology and Hepatology, Erasmus MC, University Medical Centre Rotterdam, the Netherlands

[4] Institute for Medical Technology Assessment, Erasmus University Rotterdam, the Netherlands

[5] Department of Biostatistics, Erasmus MC, University Medical Centre Rotterdam, the Netherlands

[6] Department of Paediatric Endocrinology, Erasmus MC - Sophia Children's Hospital, University Medical Centre Rotterdam, the Netherlands

**Corresponding author:**

John Vlot, MD, PhD

Erasmus MC - Sophia Children's Hospital

Department of Paediatric Surgery and Intensive Care Children

Internal postal address: room Sk-1268

P.O. Box 2040, 3000 CA Rotterdam

The Netherlands

Phone: +31 10 703 62 40, Fax: +31 10 703 68 02

john.vlot@erasmusmc.nl

**Word count:** 4000 words

**Key words:** oesophageal atresia, intralesional steroid injections, anastomotic strictures



For peer review only - http://bmjopen.bmj.com/site/about/guidelines.xhtml

For peer review only

# ABSTRACT

**Introduction**

Anastomotic stricture formation is the most common postoperative complication after oesophageal atresia repair. The standard of care is endoscopic dilatation. A possible adjuvant treatment is intralesional steroid injection, which is thought to inhibit scar tissue formation and thereby to prevent stricture recurrence. We hypothesize that this intervention could prevent refractory strictures and reduce the total number of dilatations needed in these children.

**Methods and analysis**

This is an international multicentre randomised controlled trial. Children with oesophageal atresia type C (n=110) will be randomised into intralesional steroid injection followed by balloon dilatation or dilatation only. Randomisation and intervention will take place when a third dilatation is performed. The indication for dilatation will be confirmed with an oesophagram. One radiologist – blinded for randomisation – will review all oesophagrams. The primary outcome parameter is the total number of dilatations needed with <28 days interval, which will be analysed with a linear-by-linear chi-square association test. Secondary outcome parameters include the level of dysphagia, the luminal oesophageal diameter and stricture length (measured on the oesophagrams), the influence of co-medication on stricture formation, systemic effects of intralesional steroids (cortisol levels, length and weight) and the cost-effectiveness. Patients will undergo a second oesophagram, length and weight will be measured repeatedly, a scalp hair sample will be collected and three questionnaires will be administered. The follow-up period will be six months, with evaluation 2-3 weeks, 3 months and 6 months after the intervention.

**Ethics and dissemination**

Patients will be included after written parental informed consent. The risks and burden associated with this trial are minimal. The Institutional Review Board of the Erasmus Medical Centre approved this protocol (MEC-2018-1586/NL65364.078.18). The results of the trial will be published in a peer-reviewed scientific journal and presented at international conferences.

**Trial registration**

EudraCT: 2018-002863-24; the Netherlands National Trial Register: NTR7726/NL7484.




For peer review only - http://bmjopen.bmj.com/site/about/guidelines.xhtml

# ARTICLE SUMMARY

**Strengths and limitations of this study**

- This is the first prospective study investigating the effectiveness of intralesional steroid injection combined with endoscopic dilatation for the treatment of recurrent oesophageal strictures in children operated on for oesophageal atresia (OA).
- The determination of long term cortisol levels over a period of three months will give objective information on possible systemic effects of intralesional steroid injection in children with OA.
- Besides the effectiveness and safety of intralesional steroid injection, cost-effectiveness of this treatment will be evaluated.
- The rarity of the disease makes patient recruitment challenging.

For peer review only

# INTRODUCTION

Oesophageal atresia (OA) is a congenital malformation which can present with or without a tracheoesophageal fistula, with a European prevalence of 2.43 cases per 10,000 births.[1, 2] With better treatments, survival rates have increased to over 90%.[1, 3, 4] Still, anastomotic stricture formation remains the most frequent postoperative complication in up to 60% of cases.[5] Especially refractory strictures form a great burden for both patients and their parents.

The incidence of refractory strictures is poorly reported, due to the variety in definitions used in literature. Regarded as a consensus among experts, the European Society for Paediatric Gastroenterology Hepatology and Nutrition (ESPGHAN) has set the following definition for a refractory oesophageal stricture: an anatomic restriction without endoscopic inflammation that results in dysphagia after a minimum of five dilatation procedures at maximally 4-week intervals.[6, 7]

Recently, the Dutch Consortium for Esophageal Atresia (DCEA) conducted a retrospective multicentre study in the Netherlands to assess risk factors for stricture formation in children with OA. The study population consisted of 436 children born with OA between 1999 and 2013 with an end-to-end oesophageal anastomosis. Thirty-two (7.3%) of them required ≥5 dilatations within an interval of 28 days.[5]

The initial treatment of an anastomotic stricture consists of endoscopic dilatation, either balloon dilatation or semi-rigid dilatation.[6] Consensus on the preferred technique has not yet been established. A refractory stricture requires multiple dilatations under general anaesthesia, for which the child needs to be hospitalized. This adds significantly to the burden of the disease. It is therefore important to minimize the occurrence of refractory strictures and with that the need for dilatations.

In a recent ESPGHAN guideline, various adjuvant treatments are mentioned, e.g. intralesional or systemic steroids, topical mitomycin C, oesophageal stents and surgical resection. The STEPS-EA trial will focus on intralesional steroid injections since we as well as the other centers involved in this trial have had successful results with this treatment in several patients.[8]

The literature on intralesional steroid injection in children with OA is scarce, but promising results have been described in both children and adults with all types of oesophageal strictures (Table 1). Most studies are outdated case reports or series.[9-14] Four relatively recent randomised controlled trials (RCTs) on this topic only included adults, and with other underlying diagnoses than OA, like caustic strictures after acid ingestion, peptic strictures and anastomotic strictures after oesophagectomy with gastric tube reconstruction.[15-18] Reported beneficial effects were reduction of dilatation procedures[16], longer intervals between dilatation procedures[17], improvement of luminal diameter[15] and relief of dysphagia.[18]



For peer review only - http://bmjopen.bmj.com/site/about/guidelines.xhtml

**Table 1: Summary of literature on clinical findings on intralesional steroid injections for oesophageal strictures, including retrospective cohort studies in children <12 years (range 0-14 years).**

| Author, year | Type of study | Characteristics | Main outcomes |
|---|---|---|---|
| Camargo, 2003[15] | Double-blind RCT | 14 adult patients, corrosive strictures | • No significant difference in dilatation frequency and dysphagia.<br>• Significant improvement in obtained diameter (p<0.05).<br>• No adverse events reported. |
| Ramage, 2005[16] | Double-blind RCT | 30 adult patients, peptic strictures | • Less patients required repeat dilatation in the steroid group (13% vs. 60%, p=0.0209).<br>• Shorter time to repeat dilatation in control group (p=0.01).<br>• No adverse events reported. |
| Hirdes, 2013[17] | Double-blind RCT | 60 adult patients, anastomotic strictures after oesophagectomy with gastric tube reconstruction | • No significant decrease in frequency of repeat dilatation or prolongation of dysphagia-free period.<br>• Four patients developed Candida oesophagitis. |
| Pereira-Lima, 2015[18] | Double-blind RCT | 19 adult patients, anastomotic strictures after oesophagectomy with gastric tube reconstruction | • Significant improvement on dysphagia at 1 and 6 months (p=0.021, p=0.009).<br>• No perforation, haemorrhage of oesophageal candidiasis. No other adverse events reported. |
| Kochar, 2002[19] | Prospective | 71 patients (13-78 year), all kinds of strictures | • Periodic dilatation index decreased significantly after injection (p<0.001).<br>• No adverse events reported. |
| Nijhawan, 2016[20] | Prospective | 11 adult patients, corrosive strictures | • Significant improvement of maximum dilatation (p<0.001) and number of dilatations per month (p<0.001).<br>• No adverse events reported. |
| Divarci, 2016[21] | Retrospective | 32 children (mean age 3.6 year), corrosive strictures | • Mean number of dilatation sessions was decreased (p=0.003).<br>• Mean frequency of dilatations in weeks extended (p<0.001).<br>• Only a positive effect in short segment strictures (<3 cm, 92% of patients dysphagia-free).<br>• No serious adverse events reported. One transient cushingoid phenotype, but no real adrenal suppression. |
| Cakmak, 2016[22] | Retrospective | 38 children (median age 1.5 year), EA (n=19) and corrosive strictures (n=19) | • No significant difference in treatment effectiveness between steroid injection and others (p>0.05).<br>• Intralesional steroid injections only performed in patients with long (>5 cm) and corrosive strictures & ≥5 dilatations.<br>• Four patients with oesophageal perforation, at other dilatation sessions than the intralesional steroid injection. |

For peer review only

In children, only retrospective studies have been performed, which mostly included caustic strictures.[21, 22] Divarci et al. analysed data of 32 children with corrosive strictures with a mean age of 3.6 years (± 2.5 years); after the intervention the number of dilatations had significantly decreased and the intervals between dilatations had extended.[21] Cakmak et al. included 38 children with either OA or corrosive strictures with a median age of 1.5 years (range 0-14 years) [22] but did not find a significant difference in treatment effectiveness. None of the above-mentioned studies reported any systemic effects of the local intralesional steroid injections.

All studies used triamcinolone acetonide (TAC). The exact mechanism by which TAC enhances the efficacy of dilatation is unclear. It has been proven very effective in the treatment of hypertrophic scars of the skin and keloid. A recurring anastomotic stricture can be seen as a hypertrophic lesion. The injected TAC inhibits collagen formation, enhances collagen breakdown, decreases fibrotic healing that occurs after dilatation and prevents cross-linking of collagen that causes contractions in scar tissue.[23, 24]

The recurrent dilatations and readmissions impose a substantial burden on the health care system. To date, there is no evidence on the cost-effectiveness of intralesional steroid injections. However, in the current era of evidence-based and cost-effective medicine, proof of cost-effectiveness is highly relevant.

The primary objective is to evaluate whether intralesional steroid injections combined with endoscopic dilatation can prevent refractory strictures in children with OA and recurrent oesophageal stenosis, and thus can minimize the number of dilatations needed with a 28 days interval between the dilatations.

The secondary objectives are:

- to compare the level of dysphagia and the child's eating behaviour between the two groups;
- to compare the effect of intralesional steroid injections on the luminal diameter and the stricture length between the two groups;
- to evaluate a possible influence of co-medication (e.g. antacids) on stricture formation;
- to analyse the possible systemic effects of a one-time intralesional steroid injection;
- to analyse the cost-effectiveness of the use of intralesional steroid injections to prevent refractory strictures.

# METHODS AND ANALYSIS

The STEPS-EA trial is an international, multicentre, single-blinded randomised controlled trial with a 1:1 randomisation to injection with 10 mg/ml TAC (Kenacort-A 10) prior to balloon dilatation and balloon dilatation without any injection. The participating centres are tertiary (academic) hospitals that collaborate



For peer review only - http://bmjopen.bmj.com/site/about/guidelines.xhtml

within the European Reference Network on Inherited and Congenital Abnormalities (ERNICA, ern-ernica.eu) and that routinely provide care for children with OA.

**Patient and public involvement**

Parents of patients were involved in the end-stage of the design of the trial. We presented our plans for the trial to a DCEA meeting in which also representatives of the patients association VOKS (*Vereniging voor Ouderen en Kinderen met een Slokdarmafsluiting*) took part. They were invited to comment on the study design, intervention or time required to participate in this trial. Consensus was reached on the final design during this meeting. The patients association will not be involved in the recruitment and conduct of the trial. We will involve them in dissemination, however, by presenting the trial results at a members' day or in their monthly newsletter. As the VOKS is a member of the European Federation of Esophageal Atresia and Tracheoesophageal fistula Support Groups E.V. (EAT), patients and parents throughout Europe will be informed about the results.

**Participants**

Children with OA type C[25] who underwent surgery with primary anastomosis within the first days of life and who developed a recurrent oesophageal stricture, will be eligible. Children will be included if they are ≥3 months old at the time of the intervention and in need of a third dilatation. Written parental informed consent will be obtained by the local principal investigator (PI) or another member of the local research team. Exclusion criteria are lack of parental consent or an impossibility – known from previous dilatations – to use an endoscope with a large enough diameter working-channel to pass the endoscopic injector.

**Sample size calculation**

The power calculation is based on a linear-by-linear chi-square association test comparing the total number of dilatations required within the study period (all strictures) and within a 28-days interval (refractory strictures) between the two treatment groups. The total number of dilatations will be categorized into the categories 3, 4, 5, 6, 7-10 and >10 dilatations; and a separate category for patients who are not dysphagia-free at the end of the follow-up period.

For this power calculation, we used data of the original dataset of our retrospective study in the Netherlands.[5] We selected patients from this dataset who underwent at least three dilatations with a 28 days interval (n=53). The in the retrospective study observed numbers of patients and the relative frequencies for each category are listed in Table 2. We assumed that the use of intralesional steroid injections combined with endoscopic dilatation will reduce the total number of dilatations by 50%. Note that this 50% reduction only applies to dilatations after the third dilatation, and therefore no change in the number of dilatations is assumed for the first three dilatations. This assumption leads to a different distribution for the number of dilatations within the categories, which is shown as the assumed relative frequencies for the



For peer review only - http://bmjopen.bmj.com/site/about/guidelines.xhtml

steroid group in the final column of Table 2. The details of the calculation are provided in Supplementary File 1.

In a simulation model, the required sample size to obtain a power of 80% (with a two-sided significance level of 0.05) was calculated as 52 patients per group, thus 104 in total. To account for the effects of dropout and missing data, we aim to include a total of 110 patients.

**Table 2: Assumed relative frequencies of the number of dilatations in the control and steroid groups.**

| Number of dilatations within 28 days interval | Observed number of patients (n=407)[5] | Relative frequencies control group | Assumed relative frequencies steroid group |
|---|---|---|---|
| 3 dilatations | 4 | 0.075 | 0.142 |
| 4 dilatations | 7 | 0.132 | 0.302 |
| 5 dilatations | 9 | 0.170 | 0.170 |
| 6 dilatations | 7 | 0.132 | 0.160 |
| 7-10 dilatations | 16 | 0.302 | 0.132 |
| >10 dilatations | 10 | 0.189 | 0.094 |
| Total (all numbers of dilatations combined) | 53 | 1.000 | 1.000 |

**Recruitment**

Patients will be recruited from hospitals in various European countries. Up till now, hospitals in Denmark, United Kingdom, Finland, France, Italy, and Sweden have agreed to participate. During the inclusion period it will remain possible for other centres to join. To achieve adequate participant enrolment, we have minimalized the exclusion criteria. Collaboration within ERNICA should make patient accrual achievable despite the rarity of the disease.

**Randomisation, blinding and treatment allocation**

Randomisation will be conducted via ALEA (FormsVision B.V. / ALEA Clinica B.V.), a validated software program. To achieve equal distribution of the intervention among the participating sites, block randomisation stratified per centre will be carried out. The software was prepared by an independent statistician who is not otherwise involved in the study. After inclusion, the local PI will enter the patient in ALEA and will thereupon receive an email stating the allocated treatment.

Randomization will be blinded for the radiologist who will review all oesophagrams. The control group will not receive sham treatment. The steroid that will be used, Kenacort-A 10 (see below), is a white suspension, which complicates creating a placebo. Adding excipients to normal saline is undesirable, considering the unknown effect on the healing process of the stricture. Moreover, it is deemed undesirable to inject an infant with a fluid with an unknown effect.



For peer review only - http://bmjopen.bmj.com/site/about/guidelines.xhtml

For peer review only

**Investigational product**

The intervention will be a one-time endoscopic injection of 0.25 mL Kenacort-A 10 (Bristol-Myers Squibb BV, Utrecht, the Netherlands) in each quadrant of the stricture prior to the third endoscopic dilatation. Thus, in total, 1 mL (10 mg/ml TAC, equals 12.5 mg prednisone) will be injected. During the study period, none of the patients will receive a second injection.

Kenacort-A 10 will be prepared, labelled and distributed by the trial pharmacy of the coordinating hospital. It will be delivered to the local pharmacies by courier, and the local pharmacy will deliver it to the operation room when needed. After the trial, a specific procedure for destruction of the remaining drugs is not needed; they can be disposed of locally.

**Patient timeline**

Figure 1 presents a flowchart for this study and the study procedures. The treating physician will decide on a third dilatation on the basis of the clinical signs of dysphagia and the findings on the oesophagram (thoracic x-ray with contrast, anterior-posterior and lateral). Clinical signs of dysphagia are defined as the inability to be fed age-appropriately. Findings on the oesophagram indicating a stricture are defined as a significant narrowing of the lumen, seen as a waist in the contrast on the x-ray. The treating physician can be a paediatric surgeon or paediatric gastroenterologist, depending on the local agreements in the different countries. After parental informed consent, the patient will be included and randomised to one of the study arms.

Prior to the balloon dilatation, an endoscopic needle (DVI-23-MH varices injector or equivalent) is pre-filled with 1 mL Kenacort-A 10 and passed through the endoscope. Under direct vision, 0.25 mL will be injected in each of the four quadrants of the circular stricture. After the injection needle has been retracted from the working channel and good visualization has been re-established, balloon dilatation will be performed up to the desired diameter. The balloon will remain insufflated for one minute.

During a follow up period of six months, patients will not receive any (additional) steroid injections, only balloon dilatations if needed. Meaning, patients in the steroid group will only receive one injection, and patients in the control group will receive no injections at all. After the study period has ended, treatment is again free of choice.

Two to three weeks after the dilatation procedure, a second oesophagram will be made in children in both study groups. All oesophagrams will be reviewed by one specialized paediatric radiologist of the coordinating hospital, who will determine the oesophageal diameter and stricture length. Earlier studies have proven that these measurements can be obtained from an oesophagram.[26] The parents will be informed about the results and the normal standard of care will be continued.

Each patient will be followed up for six months after the third dilatation, with recording of possible side effects, complications and additional dilatations. Length and weight will be measured at 2-3 weeks, 3 months and 6 months after the third dilatation. In the context of the cost-effectiveness analysis, the parents



For peer review only - http://bmjopen.bmj.com/site/about/guidelines.xhtml

will be asked to fill out a modified version of the iMTA Productivity Cost Questionnaire (iPCQ)[27] at 3 months after the third dilatation.

At evaluation at 6 months, we will collect a scalp hair sample from the child to determine long-term cortisone and cortisol levels.[28, 29] The hair locks will be stored for final batch analysis. Last, the parents will be asked to fill out the Montreal Feeding Scale (MFS)[30-32] and again the iPCQ. After this, the study period has ended.

**Outcome parameters**

The primary outcome parameter is the total number of dilatations required per patient with a 28 days interval between the dilatations during the study period of six months, which is defined as the period from the day of the third dilatation until six months later.

The secondary outcome parameters are:

1) Total number of dilatations within the study period, regardless of the interval.
2) Interval (in weeks) between the start of the study and the last dilatation procedure within the study period.
3) Score on the MFS.
4) The change in maximal luminal diameter after the third dilatation relative to the diameter before the third dilatation: relative change in luminal diameter = (maximal diameter after – maximal diameter before) / maximal diameter before. The diameter will be measured at the narrowest point of the oesophagus.
5) The change in length of the oesophageal stricture after the third dilatation relative to the length before the third dilatation: relative change in stricture length = (stricture length after – stricture length before) / stricture length before. The length will be measured between the two points where the oesophageal diameter starts narrowing.
6) The use of co-medication (e.g. antacids) during the study period.
7) The mean hair cortisol levels in the first three months after the third dilatation. Cortisol levels will be adjusted for age and sex.
8) Delta length standard deviation scores (SDS) and delta weight SDS between the third dilatation (intervention) and 3 and 6 months after the third dilatation.
9) Total costs of the treatment, including medical and non-medical costs.
10) Incremental costs per refractory stricture prevented and incremental costs per additional dysphagia-free patient.

**Data collection**

All participating centres are familiar with the procedure of injecting Kenacort-A 10 in the lesion via an injection needle through the endoscope. However, to guarantee equality of the intervention, the relevant



For peer review only - http://bmjopen.bmj.com/site/about/guidelines.xhtml

practitioner(s) in all centres will be trained by the PI of the trial. Radiologic interobserver variability will be avoided by having all oesophagrams reviewed by one radiologist.

The internationally validated MFS[30-32] will be used to measure dysphagia. The MFS has often been used in previous research in children with OA. The scalp hair sample will be taken from the posterior vertex; cortisol levels will be determined with the liquid chromatography-tandem mass spectrometry (LC-MS/MS) method for quantification of steroids.[28]

The cost-effectiveness analysis will follow established methods for economic evaluations and costing studies in health care.[33, 34] Both medical and non-medical costs will be collected. Medical costs will include costs of surgeries (dilatations), steroid injections, hospital days (on the ward or the intensive care unit), medication (such as analgesics) and diagnostic radiography. Costs of health care provided by others than the participating centres (such as other hospitals or general practitioners) will be ignored in this study, as these are unlikely to be affected by the intervention. Non-medical costs will include costs of special diets, costs related to hospital visits and productivity losses related to both paid and unpaid work. The non-medical costs will be measured using the iPCQ[27], supplemented with additional questions on costs of special diets and costs related to the child's hospitalisation. The original iPCQ questions are validated in English. The complete questionnaire including the additional questions will be translated to the languages required for this trial using the forward-backward translation method, as will the MFS for the languages it has not been validated for.

**Statistical analysis**

Since a standardized treatment protocol will be adopted in all centres, statistical adjustment for centre effects is considered unnecessary.

The primary outcome parameter will be analysed with a linear-by-linear chi-square association test. The total number of dilatations required with a 28 days interval (i.e. refractory strictures) within the study period of 6 months per patient will be categorized and compared between treatment groups. In case of death during the follow-up period, the outcome will be set to the highest (i.e. most severe) category. In case of drop-out during the follow-up period due to other causes (e.g. emigration, withdrawal), the subject will be excluded from the study.

The analyses for the secondary study parameters are as follows:

1) The total number of dilatations required within the study period (regardless of the interval, i.e. all strictures) will be categorized and compared between treatment groups with a linear-by-linear chi-square association test.
2) The interval (in weeks) until the patient is dysphagia-free will be compared between groups with the log-rank test and with Cox proportional hazards regression, with adjustment for treatment group and factors such as age, sex, diagnostic information and described risk factors for stricture formation like



For peer review only - http://bmjopen.bmj.com/site/about/guidelines.xhtml

anastomotic leaking and thoracoscopic repair.[5] Patients who do not become dysphagia-free during follow-up are treated as censored at the end of the follow-up period.

3) The scores on the MFS (reflecting the level of dysphagia and the eating behaviour) will be compared between study groups with a Mann-Whitney test.
4) The relative change in the oesophageal diameter and that in the length of the oesophageal stricture will be compared between study groups with an analysis of covariance model. The dependent variables in this model will be the log-transformed oesophageal diameter and length after third dilatation, and the independent variables the treatment group and the log-transformed oesophageal diameter and length before third dilatation.
6) The effect of co-medication on the primary study outcome will be assessed using a stratified Mann-Whitney test with stratification for the treatment group.
7) The mean cortisol level over the first three months after the third dilatation will be compared between study groups with a linear regression model with adjustment for age and sex.

No missing data are expected for the independent variables in the analysis of covariance models and the Cox proportional hazards regression models. In case of missing data for any of the outcomes, a complete case analysis (i.e. exclusion of the subjects who dropped out during the follow-up period) will be performed for the corresponding outcome.

**Cost-effectiveness analysis**

All medical and non-medical costs will be summated for each individual patient. Regarding the patient outcomes, the number of refractory strictures prevented and the number of dysphagia-free patients will be considered. Incremental cost-effectiveness ratios will be calculated, expressed as incremental costs per refractory stricture prevented and incremental costs per additional dysphagia-free patient. Analysis of uncertainty will be illustrated through cost-effectiveness planes (via bootstrapping).

As this RCT is done in an international setting, it must be recognized that the results may differ between countries because health care systems, treatment patterns and prices may vary. Therefore, country-level information will be collected. Data (especially resource quantities and cost prices) will be collected in all countries, and results will also be reported for each country separately. Next, a pooled summary calculation of the intervention's cost-effectiveness will be made, converting all costs into a common currency base (i.e. Euros).

# ETHICS AND DISSEMINATION

**Ethical consideration**

This study protocol was approved by the Institutional Review Board (IRB) of the Erasmus Medical Centre (MEC-2018-1586/NL65364.078.18). In case of any modifications of the protocol, a formal amendment will



For peer review only - http://bmjopen.bmj.com/site/about/guidelines.xhtml

be submitted to the IRB. Approved changes will be communicated to all relevant parties according to the rules of the IRB. The protocol has currently been submitted for local ethical approval in Amsterdam (the Netherlands), Odense (Denmark), Copenhagen (Denmark), Stockholm (Sweden), Rome (Italy) and Padua (Italy). Nijmegen (the Netherlands) and Helsinki (Finland) have already obtained local ethical approval and joined the study. To guarantee the respect of ethical rules and standard of care in all participating centres, the protocol was reviewed by the two chairs of the work package on OA within ERNICA. The informed consent and assent process of this trial is in line with the Good Clinical Practice guideline.[35]

Furthermore, we involved external experts in the form of a Data Safety Monitoring Board (DSMB). These experts are a paediatric surgeon, a pharmacologist and a statistician. All experts are independent of the sponsor and therefore competing interests will be avoided. The DSMB will monitor the safety of the study subjects and data. The board will meet at least three times: within one year of recruitment commencing, at the time of the planned interim analyses at 50% (n=55) of enrolment and at the conclusion of the trial.

**Adverse events and auditing**

Adverse events will be handled according to the guidelines of the IRB of the Erasmus Medical Centre. All adverse events will be registered during the study. Serious adverse events will be reported to the sponsor immediately and registered appropriately within 24 hours.

All participating sites will be audited once a year with monitoring of patient recruitment, source data verification, drug accountability and sample storage. The auditor will be independent and not involved in the study.

**Benefits and risk assessment**

The risks and burden associated with this study are minimal. Potential complications of oesophageal steroid injections include adrenal suppression, perforation, intramural infection, candida infection, mediastinitis and pleural effusion.[36] However, in previous studies no adverse events have been reported in relation to the steroid injections (see Table 1). Additionally, Kenacort-A 10 is a slow-release medicine and therefore a gradual exposure. This implies that the likelihood of acute exposure to a high dose of steroids will be minimal.

The burden of filling out the questionnaires and taking a hair sample are negligible. Filling out the MFS and the iPCQ will take maximally 30 minutes. All participants will undergo one extra oesophagram after the third dilatation procedure. The potential reduction in number of anaesthetic procedures needed for dilatations outweighs the burden and the radiation exposure of this oesophagram. Potential benefits of intralesional steroid injections are fewer dilatation procedures needed, with concomitant fewer anaesthetic procedures and hospital admissions, and less risk for perforation.



For peer review only - http://bmjopen.bmj.com/site/about/guidelines.xhtml

### Data management

All data will be handled confidentially and anonymously using OpenClinica V3.12.2 (OpenClinica LLC, USA) for data collection. The questionnaires will be conducted through an online survey using LimeSurvey (LimeSurvey GmbH, Germany) and GemsTracker (Erasmus MC, Rotterdam, the Netherlands). All patient data will be coded using a subject identification code list. The local PI safeguards the key to the code; the sponsor will have access to these codes. The local PI will only have access to the data of patients of their own centre; the sponsor will have access to the final trial dataset. All has been stated in a clinical trial site agreement, signed by all participating sites.

### Dissemination

The results of this trial will be published in an international peer-reviewed scientific journal, within one year after the end of the follow-up period of the last included patient. In addition, we aim to present the results at several international conferences, to inform health care professionals worldwide.

## TRIAL STATUS

The study has started in Rotterdam (the Netherlands) in February 2019, after ethical approval had been obtained. The first patient is included. Nijmegen (the Netherlands) has joined the study in October 2019 and Helsinki (Finland) in December 2019. At this moment, Amsterdam (the Netherlands), Odense (Denmark), Copenhagen (Denmark), Stockholm (Sweden), Rome (Italy) and Padua (Italy) are still waiting for local ethical approval. We expect to start the study in these sites the latest in 2020.

Considering the rarity of the disease, we expect to complete the inclusions and finish data collection for this study in five years.

For peer review only

## ACKNOWLEDGEMENTS

The author thank Benno Ure, paediatric surgeon and head of department of Paediatric Surgery of Hannover Medical School, and Frédéric Gottrand, paediatrician at the department of Paediatric Gastroenterology of University Hospital of Lille, for their input during the development of the trial protocol. The authors thank Joke Dunk, research nurse at the Erasmus University Medical Centre in Rotterdam, for her help with the implementation of the trial. Ko Hagoort provided editorial advice. At last, we thank all hospitals involved in ERNICA and the VOKS for their input and collaboration.

## AUTHORS CONTRIBUTIONS

RW is the initiator of the study. CK (PhD candidate), JV (paediatric surgeon), HIJ (paediatrician), KA (paediatric clinical pharmacologist), MS (gastroenterologist), MP (health economist), JR (statistician), EA (paediatric endocrinologist) and RW (paediatric surgeon) contributed in writing and designing of the study protocol. JV (principal investigator) and RW (project leader) will supervise the study. CK will coordinate the study and be responsible for data acquisition. CK, JV and JR will carry out the analyses. All authors have read and approved the final version of the study protocol and manuscript.

## FUNDING STATEMENT

This research received no specific grant from any funding agency in the public, commercial or not-for-profit sectors.

## COMPETING INTEREST STATEMENT

None declared.

## LICENSE STATEMENT

I, the Submitting Author has the right to grant and does grant on behalf of all authors of the Work (as defined in the below author licence), an exclusive licence and/or a non-exclusive licence for contributions from authors who are: i) UK Crown employees; ii) where BMJ has agreed a CC-BY licence shall apply, and/or iii) in accordance with the terms applicable for US Federal Government officers or employees acting as part of their official duties; on a worldwide, perpetual, irrevocable, royalty-free basis to BMJ Publishing Group Ltd ("BMJ") its licensees and where the relevant Journal is co-owned by BMJ to the co-owners of the Journal, to publish the Work in BMJ Open and any other BMJ products and to exploit all rights, as set out in our licence.

The Submitting Author accepts and understands that any supply made under these terms is made by BMJ to the Submitting Author unless you are acting as an employee on behalf of your employer or a postgraduate



For peer review only - http://bmjopen.bmj.com/site/about/guidelines.xhtml

For peer review only

student of an affiliated institution which is paying any applicable article publishing charge ("APC") for Open Access articles. Where the Submitting Author wishes to make the Work available on an Open Access basis (and intends to pay the relevant APC), the terms of reuse of such Open Access shall be governed by a Creative Commons licence – details of these licences and which Creative Commons licence will apply to this Work are set out in our licence referred to above.

## DATA AVAILABILIY STATEMENT

The data that support the findings of this trial will be available from the corresponding author upon reasonable request once the results are published.

For peer review only



For peer review only - http://bmjopen.bmj.com/site/about/guidelines.xhtml

# REFERENCES


1. Oddsberg J, Lu Y, Lagergren J. Aspects of esophageal atresia in a population-based setting: incidence, mortality, and cancer risk. *Pediatr Surg Int* 2012;28(3):249-57.
2. Pedersen RN, Calzolari E, Husby S, et al. Oesophageal atresia: prevalence, prenatal diagnosis and associated anomalies in 23 European regions. *Arch Dis Child* 2012;97(3):227-32.
3. Wang B, Tashiro J, Allan BJ, et al. A nationwide analysis of clinical outcomes among newborns with esophageal atresia and tracheoesophageal fistulas in the United States. *J Surg Res* 2014;190(2):604-12.
4. Sulkowski JP, Cooper JN, Lopez JJ, et al. Morbidity and mortality in patients with esophageal atresia. *Surgery* 2014;156(2):483-91.
5. Vergouwe FWT, Vlot J, H IJ, et al. Risk factors for refractory anastomotic strictures after oesophageal atresia repair: a multicentre study. *Arch Dis Child* 2018.
6. Krishnan U, Mousa H, Dall'Oglio L, et al. ESPGHAN-NASPGHAN Guidelines for the Evaluation and Treatment of Gastrointestinal and Nutritional Complications in Children With Esophageal Atresia-Tracheoesophageal Fistula. *J Pediatr Gastroenterol Nutr* 2016;63(5):550-70.
7. Tringali A, Thomson M, Dumonceau JM, et al. Pediatric gastrointestinal endoscopy: European Society of Gastrointestinal Endoscopy (ESGE) and European Society for Paediatric Gastroenterology Hepatology and Nutrition (ESPGHAN) Guideline Executive summary. *Endoscopy* 2017;49(1):83-91.
8. Ten Kate CA, Vlot J, Sloots CEJ, et al. The effect of intralesional steroid injections on esophageal strictures and the child as whole: a case series. *J Pediatr Surg* 2019.
9. Holder TM, Ashcraft KW, Leape L. The treatment of patients with esophageal strictures by local steroid injections. *J Pediatr Surg* 1969;4(6):646-53.
10. Zein NN, Greseth JM, Perrault J. Endoscopic intralesional steroid injections in the management of refractory esophageal strictures. *Gastrointest Endosc* 1995;41(6):596-8.
11. Mendelsohn HJ, Maloney WH. The treatment of benign strictures of the esophagus with cortisone injection. *Ann Otol Rhinol Laryngol* 1970;79(5):900-4.
12. Gandhi RP, Cooper A, Barlow BA. Successful management of esophageal strictures without resection or replacement. *J Pediatr Surg* 1989;24(8):745-9; discussion 49-50.
13. Kirsch M, Blue M, Desai RK, et al. Intralesional steroid injections for peptic esophageal strictures. *Gastrointest Endosc* 1991;37(2):180-2.
14. Kochhar R, Ray JD, Sriram PV, et al. Intralesional steroids augment the effects of endoscopic dilation in corrosive esophageal strictures. *Gastrointest Endosc* 1999;49(4 Pt 1):509-13.
15. Camargo MA, Lopes LR, Grangeia Tde A, et al. [Use of corticosteroids after esophageal dilations on patients with corrosive stenosis: prospective, randomized and double-blind study]




For peer review only - http://bmjopen.bmj.com/site/about/guidelines.xhtml

O uso de corticoesteroides apos dilata o esofagica em pacientes portadores de estenose por substancias corrosivas: estudo prospectivo, randomizado e duplo-cego. *Rev Assoc Med Bras (1992)* 2003;49(3):286-92.

16. Ramage JI, Jr., Rumalla A, Baron TH, et al. A prospective, randomized, double-blind, placebo-controlled trial of endoscopic steroid injection therapy for recalcitrant esophageal peptic strictures. *Am J Gastroenterol* 2005;100(11):2419-25.
17. Hirdes MM, van Hooft JE, Koornstra JJ, et al. Endoscopic corticosteroid injections do not reduce dysphagia after endoscopic dilation therapy in patients with benign esophagogastric anastomotic strictures. *Clin Gastroenterol Hepatol* 2013;11(7):795-801 e1.
18. Pereira-Lima JC, Lemos Bonotto M, Hahn GD, et al. A prospective randomized trial of intralesional triamcinolone injections after endoscopic dilation for complex esophagogastric anastomotic strictures: steroid injection after endoscopic dilation. *Surg Endosc* 2015;29(5):1156-60.
19. Kochhar R, Makharia GK. Usefulness of intralesional triamcinolone in treatment of benign esophageal strictures. *Gastrointest Endosc* 2002;56(6):829-34.
20. Nijhawan S, Udawat HP, Nagar P. Aggressive bougie dilatation and intralesional steroids is effective in refractory benign esophageal strictures secondary to corrosive ingestion. *Dis Esophagus* 2016;29(8):1027-31.
21. Divarci E, Celtik U, Dokumcu Z, et al. The Efficacy of Intralesional Steroid Injection in the Treatment of Corrosive Esophageal Strictures in Children. *Surg Laparosc Endosc Percutan Tech* 2016;26(6):e122-e25.
22. Cakmak M, Boybeyi O, Gollu G, et al. Endoscopic balloon dilatation of benign esophageal strictures in childhood: a 15-year experience. *Dis Esophagus* 2016;29(2):179-84.
23. Ashcraft KW, Holder TM. The expeimental treatment of esophageal strictures by intralesional steroid injections. *J Thorac Cardiovasc Surg* 1969;58(5):685-91 passim.
24. Ketchum LD, Smith J, Robinson DW, et al. The treatment of hypertrophic scar, keloid and scar contracture by triamcinolone acetonide. *Plast Reconstr Surg* 1966;38(3):209-18.
25. Gross RE. Atresia of the esophagus. *Am J Dis Child* 1947;74(3):369.
26. Sun LY, Laberge JM, Yousef Y, et al. The Esophageal Anastomotic Stricture Index (EASI) for the management of esophageal atresia. *J Pediatr Surg* 2015;50(1):107-10.
27. Bouwmans C, Krol M, Severens H, et al. The iMTA Productivity Cost Questionnaire: A Standardized Instrument for Measuring and Valuing Health-Related Productivity Losses. *Value Health* 2015;18(6):753-8.
28. Noppe G, de Rijke YB, Dorst K, et al. LC-MS/MS-based method for long-term steroid profiling in human scalp hair. *Clin Endocrinol (Oxf)* 2015;83(2):162-6.
29. Wester VL, Noppe G, Savas M, et al. Hair analysis reveals subtle HPA axis suppression associated with use of local corticosteroids: The Lifelines cohort study. *Psychoneuroendocrinology* 2017;80:1-6.

For peer review only



For peer review only - http://bmjopen.bmj.com/site/about/guidelines.xhtml

30. Baird R, Levesque D, Birnbaum R, et al. A pilot investigation of feeding problems in children with esophageal atresia. *Dis Esophagus* 2015;28(3):224-8.

31. Ramsay M, Martel C, Porporino M, et al. The Montreal Children's Hospital Feeding Scale: A brief bilingual screening tool for identifying feeding problems. *Paediatr Child Health* 2011;16(3):147-e17.

32. Van Dijk M, Timmerman M, Martel C, et al. Towards the development of a Dutch screening instrument for the detection of feeding problems in young children. *Netherlands Journal of Psychology* 2011;66:112-19.

33. National Health Institute Diemen. Guidelines for economic evaluations of health care [in Dutch].

34. National Health Care Institute Diemen. Manual for costing research. Methods and reference prices for economic evaluations in health care. 2015 Updated edition [in Dutch]. .

35. ICH Expert Working Group. Guideline for Good Clinical Practice. 1996.

36. Kochhar R, Poornachandra KS. Intralesional steroid injection therapy in the management of resistant gastrointestinal strictures. *World J Gastrointest Endosc* 2010;2(2):61-8.



For peer review only - http://bmjopen.bmj.com/site/about/guidelines.xhtml

# FIGURE LEGENDS

**Figure 1: Flowchart of the study design**

OA = oesophageal atresia, AP = anterior-posterior, iPCQ = iMTA Productivity Cost Questionnaire, MFS = Montreal Feeding Scale. **Bold with underline** = study procedures, the rest is standard of care.

For peer review only



For peer review only - http://bmjopen.bmj.com/site/about/guidelines.xhtml

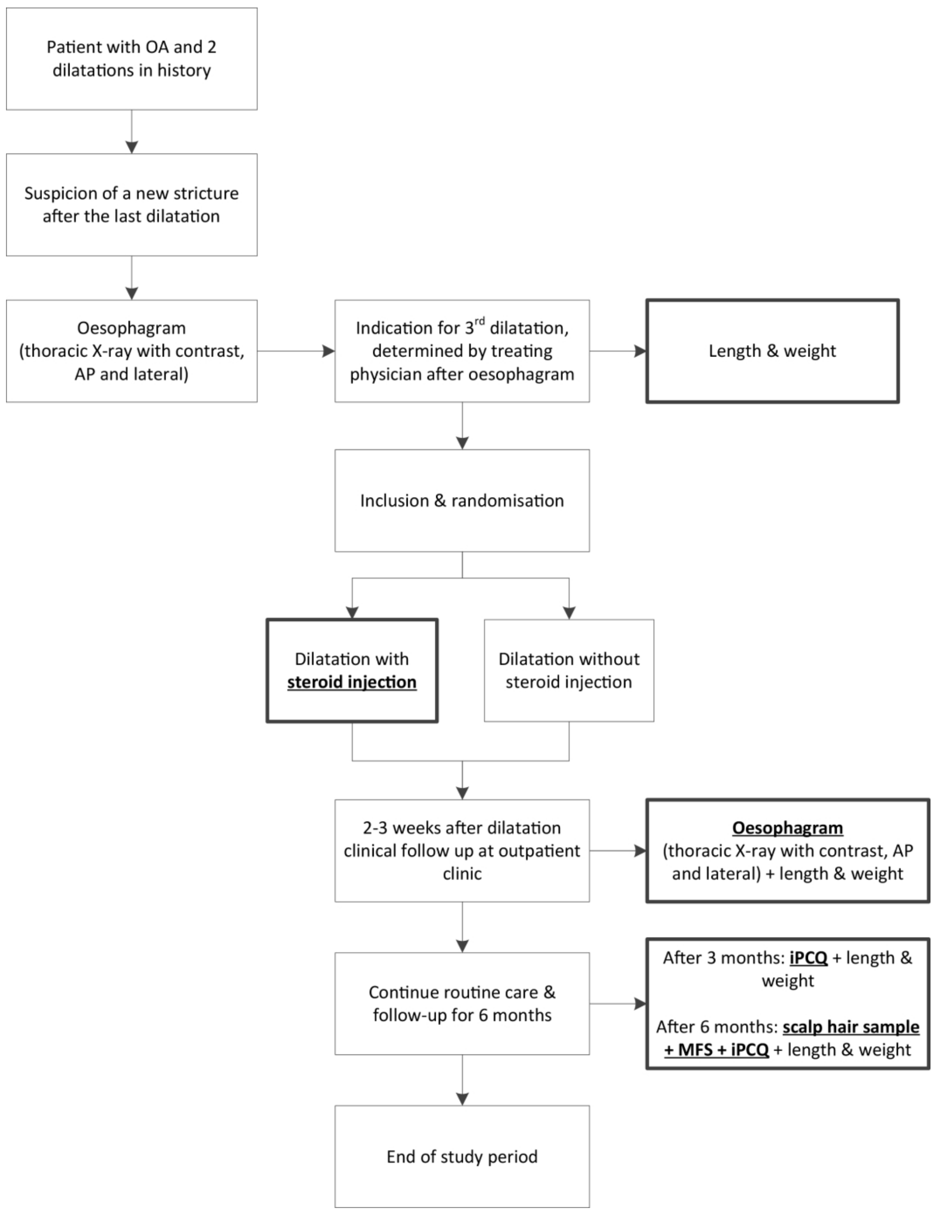


Figure 1: Flowchart of the study design
OA = oesophageal atresia, AP = anterior-posterior, iPCQ = iMTA Productivity Cost Questionnaire, MFS = Montreal Feeding Scale. Bold with underline = study procedures, the rest is standard of care.

# SUPPLEMENTARY FILE 1

To calculate the distribution of the number of dilatations in the steroid group, we assumed the following:

- The first 3 dilatations are unaffected by the intervention; thus the 4 patients with 3 dilatations in the observed data would still have 3 dilatations with steroid treatment.
- The 7 patients with 4 dilatations in the observed data would, if steroid treatment were applied, be equally divided over the categories 3 dilatations and 4 dilatations (3.5 patients in each category).
- The 9 patients with 5 dilatations in the observed data would, if steroid treatment were applied, have 4 dilatations.
- The 7 patients with 6 dilatations in the observed data would, if steroid treatment were applied, be equally divided over the categories 4 dilatations and 5 dilatations (3.5 patients in each category).
- The 16 patients with 7-10 dilatations in the observed data would, if steroid treatment were applied, be divided over the categories 5, 6 and 7-10 dilatations (patients with 7 dilatations would have 5 dilatations, patients with 8 dilatations would have 5 or 6 dilatations, patients with 9 dilatations would have 6 dilatations and patients with 10 dilatations would have 6 or 7 dilatations).
- The 10 patients with >10 dilatations in the observed data would, if steroid treatment were applied, be divided over the categories 7-10 and >10 dilatations (5 patients in each category).

These assumptions lead to the predicted numbers of patients shown in Supplementary Table 1. The relative frequency distribution in the steroid group is then calculated by dividing the predicted numbers of patients by the total number of 53 patients.

| **Number of dilatations within 28 days interval** | **Observed number of patients (n=407)** [1] | **Predicted number of patients with steroid treatment** | **Relative frequencies control group** | **Assumed relative frequencies steroid group** |
|---|---|---|---|---|
| 3 dilatations | 4 | 7.5 (4+3.5) | 0.075 | 0.142 |
| 4 dilatations | 7 | 16 (3.5+9+3.5) | 0.132 | 0.302 |
| 5 dilatations | 9 | 9 (3.5+2+3.5) | 0.170 | 0.170 |
| 6 dilatations | 7 | 8.5 (3.5+3+2) | 0.132 | 0.160 |
| 7-10 dilatations* | 16 | 7 (2+5) | 0.302 | 0.132 |
| >10 dilatations* | 10 | 5 (5) | 0.189 | 0.094 |
| Total (all numbers of dilatations combined) | 53 | 53 | 1.000 | 1.000 |

**Supplementary Table 1:** Assumed relative frequencies of the number of dilatations in the control and steroid groups, including the calculated predicted number of patients with steroid treatment. *See Supplementary Table 2 for the exact number of dilatations within these categories.

For peer review only

| Number of dilatations within 28 days interval | Observed number of patients (n=407)[1] |
|---|---|
| 3 dilatations | 4 |
| 4 dilatations | 7 |
| 5 dilatations | 9 |
| 6 dilatations | 7 |
| 7 dilatations | 2 |
| 8 dilatations | 7 |
| 9 dilatations | 3 |
| 10 dilatations | 4 |
| 12 dilatations | 2 |
| 13 dilatations | 2 |
| 15 dilatations | 1 |
| 18 dilatations | 2 |
| 24 dilatations | 1 |
| 30 dilatations | 1 |
| 34 dilatations | 1 |
| Total (all numbers of dilatations combined) | 53 |

**Supplementary Table 2:** *Exact* number of dilatations as extracted from the *original dataset* of the retrospective study in the Netherlands.[1]

1. Vergouwe FWT, Vlot J, H IJ, et al. Risk factors for refractory anastomotic strictures after oesophageal atresia repair: a multicentre study. *Arch Dis Child* 2018.

For peer review only

# Reporting checklist for protocol of a clinical trial.

Based on the SPIRIT guidelines.

## Instructions to authors

Complete this checklist by entering the page numbers from your manuscript where readers will find each of the items listed below.

Your article may not currently address all the items on the checklist. Please modify your text to include the missing information. If you are certain that an item does not apply, please write "n/a" and provide a short explanation.

Upload your completed checklist as an extra file when you submit to a journal.

In your methods section, say that you used the SPIRITreporting guidelines, and cite them as:

Chan A-W, Tetzlaff JM, Altman DG, Laupacis A, Gøtzsche PC, Krleža-Jerić K, Hróbjartsson A, Mann H, Dickersin K, Berlin J, Doré C, Parulekar W, Summerskill W, Groves T, Schulz K, Sox H, Rockhold FW, Rennie D, Moher D. SPIRIT 2013 Statement: Defining standard protocol items for clinical trials. Ann Intern Med. 2013;158(3):200-207

| | | Reporting Item | Page Number |
|---|---|---|---|
| **Administrative information** | | | |
| Title | #1 | Descriptive title identifying the study design, population, interventions, and, if applicable, trial acronym | 1 |
| Trial registration | #2a | Trial identifier and registry name. If not yet registered, name of intended registry | 2 |
| Trial registration: data set | #2b | All items from the World Health Organization Trial Registration Data Set | 2 |
| Protocol version | #3 | Date and version identifier | 2 |
| Funding | #4 | Sources and types of financial, material, and other support | 15 |
| Roles and responsibilities: contributorship | #5a | Names, affiliations, and roles of protocol contributors | 15 |

For peer review only

| | | | |
|---|---|---|---|
| Roles and responsibilities: sponsor contact information | #5b | Name and contact information for the trial sponsor | 1 |
| Roles and responsibilities: sponsor and funder | #5c | Role of study sponsor and funders, if any, in study design; collection, management, analysis, and interpretation of data; writing of the report; and the decision to submit the report for publication, including whether they will have ultimate authority over any of these activities | 15 |
| Roles and responsibilities: committees | #5d | Composition, roles, and responsibilities of the coordinating centre, steering committee, endpoint adjudication committee, data management team, and other individuals or groups overseeing the trial, if applicable (see Item 21a for data monitoring committee) | 15 |
| **Introduction** | | | |
| Background and rationale | #6a | Description of research question and justification for undertaking the trial, including summary of relevant studies (published and unpublished) examining benefits and harms for each intervention | 4-6 |
| Background and rationale: choice of comparators | #6b | Explanation for choice of comparators | 4-6 |
| Objectives | #7 | Specific objectives or hypotheses | 6 |
| Trial design | #8 | Description of trial design including type of trial (eg, parallel group, crossover, factorial, single group), allocation ratio, and framework (eg, superiority, equivalence, non-inferiority, exploratory) | 6-7 |
| **Methods: Participants, interventions, and outcomes** | | | |
| Study setting | #9 | Description of study settings (eg, community clinic, academic hospital) and list of countries where data will be collected. Reference to where list of study sites can be obtained | 6-7 |
| Eligibility criteria | #10 | Inclusion and exclusion criteria for participants. If applicable, eligibility criteria for study centres and individuals who will | 7 |

For peer review only

| | | | |
|---|---|---|---|
| | | perform the interventions (eg, surgeons, psychotherapists) | |
| Interventions: description | #11a | Interventions for each group with sufficient detail to allow replication, including how and when they will be administered | 9 |
| Interventions: modifications | #11b | Criteria for discontinuing or modifying allocated interventions for a given trial participant (eg, drug dose change in response to harms, participant request, or improving / worsening disease) | N/A |
| Interventions: adherance | #11c | Strategies to improve adherence to intervention protocols, and any procedures for monitoring adherence (eg, drug tablet return; laboratory tests) | N/A |
| Interventions: concomitant care | #11d | Relevant concomitant care and interventions that are permitted or prohibited during the trial | 9 |
| Outcomes | #12 | Primary, secondary, and other outcomes, including the specific measurement variable (eg, systolic blood pressure), analysis metric (eg, change from baseline, final value, time to event), method of aggregation (eg, median, proportion), and time point for each outcome. Explanation of the clinical relevance of chosen efficacy and harm outcomes is strongly recommended | 10 |
| Participant timeline | #13 | Time schedule of enrolment, interventions (including any run-ins and washouts), assessments, and visits for participants. A schematic diagram is highly recommended (see Figure) | 9 |
| Sample size | #14 | Estimated number of participants needed to achieve study objectives and how it was determined, including clinical and statistical assumptions supporting any sample size calculations | 7-8 |
| Recruitment | #15 | Strategies for achieving adequate participant enrolment to reach target sample size | 8 |
| **Methods: Assignment of interventions (for controlled trials)** | | | |
| Allocation: sequence generation | #16a | Method of generating the allocation sequence (eg, computer-generated random numbers), and list of any factors for stratification. To reduce predictability of a random sequence, details of any planned restriction (eg, blocking) should be provided in a separate document that is unavailable to those who enrol participants or assign interventions | 8 |

For peer review only

| | | | |
|---|---|---|---|
| Allocation concealment mechanism | #16b | Mechanism of implementing the allocation sequence (eg, central telephone; sequentially numbered, opaque, sealed envelopes), describing any steps to conceal the sequence until interventions are assigned | 8 |
| Allocation: implementation | #16c | Who will generate the allocation sequence, who will enrol participants, and who will assign participants to interventions | 8 |
| Blinding (masking) | #17a | Who will be blinded after assignment to interventions (eg, trial participants, care providers, outcome assessors, data analysts), and how | 8 |
| Blinding (masking): emergency unblinding | #17b | If blinded, circumstances under which unblinding is permissible, and procedure for revealing a participant's allocated intervention during the trial | N/A |
| **Methods: Data collection, management, and analysis** | | | |
| Data collection plan | #18a | Plans for assessment and collection of outcome, baseline, and other trial data, including any related processes to promote data quality (eg, duplicate measurements, training of assessors) and a description of study instruments (eg, questionnaires, laboratory tests) along with their reliability and validity, if known. Reference to where data collection forms can be found, if not in the protocol | 10-11 |
| Data collection plan: retention | #18b | Plans to promote participant retention and complete follow-up, including list of any outcome data to be collected for participants who discontinue or deviate from intervention protocols | 10-11 |
| Data management | #19 | Plans for data entry, coding, security, and storage, including any related processes to promote data quality (eg, double data entry; range checks for data values). Reference to where details of data management procedures can be found, if not in the protocol | 10-11 |
| Statistics: outcomes | #20a | Statistical methods for analysing primary and secondary outcomes. Reference to where other details of the statistical analysis plan can be found, if not in the protocol | 11-12 |
| Statistics: additional analyses | #20b | Methods for any additional analyses (eg, subgroup and adjusted analyses) | 11-12 |

For peer review only

| | | | |
|---|---|---|---|
| Statistics: analysis population and missing data | #20c | Definition of analysis population relating to protocol non-adherence (eg, as randomised analysis), and any statistical methods to handle missing data (eg, multiple imputation) | 11-12 |
| **Methods: Monitoring** | | | |
| Data monitoring: formal committee | #21a | Composition of data monitoring committee (DMC); summary of its role and reporting structure; statement of whether it is independent from the sponsor and competing interests; and reference to where further details about its charter can be found, if not in the protocol. Alternatively, an explanation of why a DMC is not needed | 13 |
| Data monitoring: interim analysis | #21b | Description of any interim analyses and stopping guidelines, including who will have access to these interim results and make the final decision to terminate the trial | 13 |
| Harms | #22 | Plans for collecting, assessing, reporting, and managing solicited and spontaneously reported adverse events and other unintended effects of trial interventions or trial conduct | 13 |
| Auditing | #23 | Frequency and procedures for auditing trial conduct, if any, and whether the process will be independent from investigators and the sponsor | 13 |
| **Ethics and dissemination** | | | |
| Research ethics approval | #24 | Plans for seeking research ethics committee / institutional review board (REC / IRB) approval | 12 |
| Protocol amendments | #25 | Plans for communicating important protocol modifications (eg, changes to eligibility criteria, outcomes, analyses) to relevant parties (eg, investigators, REC / IRBs, trial participants, trial registries, journals, regulators) | 12 |
| Consent or assent | #26a | Who will obtain informed consent or assent from potential trial participants or authorised surrogates, and how (see Item 32) | 7 |
| Consent or assent: ancillary studies | #26b | Additional consent provisions for collection and use of participant data and biological specimens in ancillary studies, if applicable | N/A |
| Confidentiality | #27 | How personal information about potential and enrolled participants will be collected, shared, and maintained in order to protect confidentiality before, during, and after the trial | 10-11 |

For peer review only

| | | | |
|---|---|---|---|
| Declaration of interests | #28 | Financial and other competing interests for principal investigators for the overall trial and each study site | 15 |
| Data access | #29 | Statement of who will have access to the final trial dataset, and disclosure of contractual agreements that limit such access for investigators | 10-11 |
| Ancillary and post trial care | #30 | Provisions, if any, for ancillary and post-trial care, and for compensation to those who suffer harm from trial participation | N/A |
| Dissemination policy: trial results | #31a | Plans for investigators and sponsor to communicate trial results to participants, healthcare professionals, the public, and other relevant groups (eg, via publication, reporting in results databases, or other data sharing arrangements), including any publication restrictions | 14 |
| Dissemination policy: authorship | #31b | Authorship eligibility guidelines and any intended use of professional writers | 14 |
| Dissemination policy: reproducible research | #31c | Plans, if any, for granting public access to the full protocol, participant-level dataset, and statistical code | 14 |
| **Appendices** | | | |
| Informed consent materials | #32 | Model consent form and other related documentation given to participants and authorised surrogates | N/A |
| Biological specimens | #33 | Plans for collection, laboratory evaluation, and storage of biological specimens for genetic or molecular analysis in the current trial and for future use in ancillary studies, if applicable | N/A |

The SPIRIT checklist is distributed under the terms of the Creative Commons Attribution License CC-BY-ND 3.0. This checklist was completed on 15. July 2019 using https://www.goodreports.org/, a tool made by the EQUATOR Network in collaboration with Penelope.ai

For peer review only

BMJ Open

# The STEPS-EA trial: intralesional STEroid injections to Prevent refractory Strictures in patients with Esophageal Atresia – study protocol for an international, multicentre randomised controlled trial


| | |
|---|---|
| Journal: | *BMJ Open* |
| Manuscript ID | bmjopen-2019-033030.R2 |
| Article Type: | Protocol |
| Date Submitted by the Author: | 25-Nov-2019 |


| | |
|---|---|
| Complete List of Authors: | ten Kate, Chantal; Erasmus University Medical Center - Sophia Children's Hospital, Paediatric Surgery<br>Vlot, John; Erasmus University Medical Center - Sophia Children's Hospital, Paediatric Surgery<br>Ijsselstijn, Hanneke ; Erasmus University Medical Center - Sophia Children's Hospital, Paediatric Surgery<br>Allegaert, Karel; Erasmus University Medical Center - Sophia Children's Hospital, Paediatrics, Division of Neonatology<br>Spaander, Manon; Erasmus University Medical Center, Gastroenterology and Hepatology<br>Poley, Marten; Erasmus University Rotterdam, Institute for Medical Technology Assessment<br>van Rosmalen, Joost; Erasmus University Medical Center, Biostatistics<br>van den Akker, Erica; Erasmus University Medical Center - Sophia Children's Hospital, Paediatric Endocrinology<br>Wijnen, Rene; Erasmus University Medical Center - Sophia Children's Hospital, Paediatric Surgery |


| | |
|---|---|
| <b>Primary Subject Heading</b>: | Surgery |
| Secondary Subject Heading: | Paediatrics, Gastroenterology and hepatology |
| Keywords: | oesophageal atresia, intralesional steroid injections, anastomotic strictures |
| | |


SCHOLARONE™
Manuscripts

# TITLE PAGE

**Title:**

The STEPS-EA trial: intralesional STEroid injections to Prevent refractory Strictures in patients with Esophageal Atresia – study protocol for an international, multicentre randomised controlled trial

**Authors and affiliations:**

Chantal A. ten Kate, MD[1], John Vlot, MD PhD[1], Hanneke IJsselstijn, MD PhD[1], Karel Allegaert, MD PhD[2], Manon C.W. Spaander, MD PhD[3], Marten J. Poley, PhD LLM[1, 4], Joost van Rosmalen, PhD[5], Erica L.T. van den Akker, MD PhD[6], Rene M.H. Wijnen, MD PhD[1]

[1] Department of Paediatric Surgery and Intensive Care, Erasmus MC - Sophia Children's Hospital, University Medical Centre Rotterdam, the Netherlands

[2] Department of Paediatrics, Division of Neonatology, Erasmus MC - Sophia Children's Hospital, University Medical Centre Rotterdam, the Netherlands

[3] Department of Gastroenterology and Hepatology, Erasmus MC, University Medical Centre Rotterdam, the Netherlands

[4] Institute for Medical Technology Assessment, Erasmus University Rotterdam, the Netherlands

[5] Department of Biostatistics, Erasmus MC, University Medical Centre Rotterdam, the Netherlands

[6] Department of Paediatric Endocrinology, Erasmus MC - Sophia Children's Hospital, University Medical Centre Rotterdam, the Netherlands

**Corresponding author:**

John Vlot, MD, PhD

Erasmus MC - Sophia Children's Hospital

Department of Paediatric Surgery and Intensive Care Children

Internal postal address: room Sk-1268

P.O. Box 2040, 3000 CA Rotterdam

The Netherlands

Phone: +31 10 703 62 40, Fax: +31 10 703 68 02

john.vlot@erasmusmc.nl

**Word count:**

**Key words:** oesophageal atresia, intralesional steroid injections, anastomotic strictures



For peer review only - http://bmjopen.bmj.com/site/about/guidelines.xhtml

# ABSTRACT

**Introduction**

Anastomotic stricture formation is the most common postoperative complication after oesophageal atresia repair. The standard of care is endoscopic dilatation. A possible adjuvant treatment is intralesional steroid injection, which is thought to inhibit scar tissue formation and thereby to prevent stricture recurrence. We hypothesize that this intervention could prevent refractory strictures and reduce the total number of dilatations needed in these children.

**Methods and analysis**

This is an international multicentre randomised controlled trial. Children with oesophageal atresia type C (n=110) will be randomised into intralesional steroid injection followed by balloon dilatation or dilatation only. Randomisation and intervention will take place when a third dilatation is performed. The indication for dilatation will be confirmed with an oesophagram. One radiologist – blinded for randomisation – will review all oesophagrams. The primary outcome parameter is the total number of dilatations needed with <28 days interval, which will be analysed with a linear-by-linear chi-square association test. Secondary outcome parameters include the level of dysphagia, the luminal oesophageal diameter and stricture length (measured on the oesophagrams), the influence of co-medication on stricture formation, systemic effects of intralesional steroids (cortisol levels, length and weight) and the cost-effectiveness. Patients will undergo a second oesophagram, length and weight will be measured repeatedly, a scalp hair sample will be collected and three questionnaires will be administered. The follow-up period will be six months, with evaluation 2-3 weeks, 3 months and 6 months after the intervention.

**Ethics and dissemination**

Patients will be included after written parental informed consent. The risks and burden associated with this trial are minimal. The Institutional Review Board of the Erasmus Medical Centre approved this protocol (MEC-2018-1586/NL65364.078.18). The results of the trial will be published in a peer-reviewed scientific journal and presented at international conferences.

**Trial registration**

EudraCT: 2018-002863-24; the Netherlands National Trial Register: NTR7726/NL7484.


For peer review only

# ARTICLE SUMMARY

**Strengths and limitations of this study**

- This is the first prospective study investigating the effectiveness of intralesional steroid injection combined with endoscopic dilatation for the treatment of recurrent oesophageal strictures in children operated on for oesophageal atresia (OA).
- The determination of long term cortisol levels over a period of three months will give objective information on possible systemic effects of intralesional steroid injection in children with OA.
- Besides the effectiveness and safety of intralesional steroid injection, cost-effectiveness of this treatment will be evaluated.
- The rarity of the disease makes patient recruitment challenging.

For peer review only



For peer review only - http://bmjopen.bmj.com/site/about/guidelines.xhtml

# INTRODUCTION

Oesophageal atresia (OA) is a congenital malformation which can present with or without a tracheoesophageal fistula, with a European prevalence of 2.43 cases per 10,000 births.[1, 2] With better treatments, survival rates have increased to over 90%.[1, 3, 4] Still, anastomotic stricture formation remains the most frequent postoperative complication in up to 60% of cases.[5] Especially refractory strictures form a great burden for both patients and their parents.

The incidence of refractory strictures is poorly reported, due to the variety in definitions used in literature. Regarded as a consensus among experts, the European Society for Paediatric Gastroenterology Hepatology and Nutrition (ESPGHAN) has set the following definition for a refractory oesophageal stricture: an anatomic restriction without endoscopic inflammation that results in dysphagia after a minimum of five dilatation procedures at maximally 4-week intervals.[6, 7]

Recently, the Dutch Consortium for Esophageal Atresia (DCEA) conducted a retrospective multicentre study in the Netherlands to assess risk factors for stricture formation in children with OA. The study population consisted of 436 children born with OA between 1999 and 2013 with an end-to-end oesophageal anastomosis. Thirty-two (7.3%) of them required ≥5 dilatations within an interval of 28 days.[5]

The initial treatment of an anastomotic stricture consists of endoscopic dilatation, either balloon dilatation or semi-rigid dilatation.[6] Consensus on the preferred technique has not yet been established. A refractory stricture requires multiple dilatations under general anaesthesia, for which the child needs to be hospitalized. This adds significantly to the burden of the disease. It is therefore important to minimize the occurrence of refractory strictures and with that the need for dilatations.

In a recent ESPGHAN guideline, various adjuvant treatments are mentioned, e.g. intralesional or systemic steroids, topical mitomycin C, oesophageal stents and surgical resection. The STEPS-EA trial will focus on intralesional steroid injections since we as well as the other centers involved in this trial have had successful results with this treatment in several patients.[8]

The literature on intralesional steroid injection in children with OA is scarce, but promising results have been described in both children and adults with all types of oesophageal strictures (Table 1). Most studies are outdated case reports or series.[9-14] Four relatively recent randomised controlled trials (RCTs) on this topic only included adults, and with other underlying diagnoses than OA, like caustic strictures after acid ingestion, peptic strictures and anastomotic strictures after oesophagectomy with gastric tube reconstruction.[15-18] Reported beneficial effects were reduction of dilatation procedures[16], longer intervals between dilatation procedures[17], improvement of luminal diameter[15] and relief of dysphagia.[18]



For peer review only - http://bmjopen.bmj.com/site/about/guidelines.xhtml

**Table 1: Summary of literature on clinical findings on intralesional steroid injections for oesophageal strictures, including retrospective cohort studies in children <12 years (range 0-14 years).**

| Author, year | Type of study | Characteristics | Main outcomes |
|---|---|---|---|
| Camargo, 2003[15] | Double-blind RCT | 14 adult patients, corrosive strictures | • No significant difference in dilatation frequency and dysphagia.<br>• Significant improvement in obtained diameter (p<0.05).<br>• No adverse events reported. |
| Ramage, 2005[16] | Double-blind RCT | 30 adult patients, peptic strictures | • Less patients required repeat dilatation in the steroid group (13% vs. 60%, p=0.0209).<br>• Shorter time to repeat dilatation in control group (p=0.01).<br>• No adverse events reported. |
| Hirdes, 2013[17] | Double-blind RCT | 60 adult patients, anastomotic strictures after oesophagectomy with gastric tube reconstruction | • No significant decrease in frequency of repeat dilatation or prolongation of dysphagia-free period.<br>• Four patients developed Candida oesophagitis. |
| Pereira-Lima, 2015[18] | Double-blind RCT | 19 adult patients, anastomotic strictures after oesophagectomy with gastric tube reconstruction | • Significant improvement on dysphagia at 1 and 6 months (p=0.021, p=0.009).<br>• No perforation, haemorrhage of oesophageal candidiasis. No other adverse events reported. |
| Kochar, 2002[19] | Prospective | 71 patients (13-78 year), all kinds of strictures | • Periodic dilatation index decreased significantly after injection (p<0.001).<br>• No adverse events reported. |
| Nijhawan, 2016[20] | Prospective | 11 adult patients, corrosive strictures | • Significant improvement of maximum dilatation (p<0.001) and number of dilatations per month (p<0.001).<br>• No adverse events reported. |
| Divarci, 2016[21] | Retrospective | 32 children (mean age 3.6 year), corrosive strictures | • Mean number of dilatation sessions was decreased (p=0.003).<br>• Mean frequency of dilatations in weeks extended (p<0.001).<br>• Only a positive effect in short segment strictures (<3 cm, 92% of patients dysphagia-free).<br>• No serious adverse events reported. One transient cushingoid phenotype, but no real adrenal suppression. |
| Cakmak, 2016[22] | Retrospective | 38 children (median age 1.5 year), EA (n=19) and corrosive strictures (n=19) | • No significant difference in treatment effectiveness between steroid injection and others (p>0.05).<br>• Intralesional steroid injections only performed in patients with long (>5 cm) and corrosive strictures & ≥5 dilatations.<br>• Four patients with oesophageal perforation, at other dilatation sessions than the intralesional steroid injection. |



For peer review only - http://bmjopen.bmj.com/site/about/guidelines.xhtml

In children, only retrospective studies have been performed, which mostly included caustic strictures.[21, 22] Divarci et al. analysed data of 32 children with corrosive strictures with a mean age of 3.6 years (± 2.5 years); after the intervention the number of dilatations had significantly decreased and the intervals between dilatations had extended.[21] Cakmak et al. included 38 children with either OA or corrosive strictures with a median age of 1.5 years (range 0-14 years) [22] but did not find a significant difference in treatment effectiveness. None of the above-mentioned studies reported any systemic effects of the local intralesional steroid injections.

All studies used triamcinolone acetonide (TAC). The exact mechanism by which TAC enhances the efficacy of dilatation is unclear. It has been proven very effective in the treatment of hypertrophic scars of the skin and keloid. A recurring anastomotic stricture can be seen as a hypertrophic lesion. The injected TAC inhibits collagen formation, enhances collagen breakdown, decreases fibrotic healing that occurs after dilatation and prevents cross-linking of collagen that causes contractions in scar tissue.[23, 24]

The recurrent dilatations and readmissions impose a substantial burden on the health care system. To date, there is no evidence on the cost-effectiveness of intralesional steroid injections. However, in the current era of evidence-based and cost-effective medicine, proof of cost-effectiveness is highly relevant.

The primary objective is to evaluate whether intralesional steroid injections combined with endoscopic dilatation can prevent refractory strictures in children with OA and recurrent oesophageal stenosis, and thus can minimize the number of dilatations needed with a 28 days interval between the dilatations.

The secondary objectives are:

- to compare the level of dysphagia and the child's eating behaviour between the two groups;
- to compare the effect of intralesional steroid injections on the luminal diameter and the stricture length between the two groups;
- to evaluate a possible influence of co-medication (e.g. antacids) on stricture formation;
- to analyse the possible systemic effects of a one-time intralesional steroid injection;
- to analyse the cost-effectiveness of the use of intralesional steroid injections to prevent refractory strictures.

## METHODS AND ANALYSIS

The STEPS-EA trial is an international, multicentre, single-blinded randomised controlled trial with a 1:1 randomisation to injection with 10 mg/ml TAC (Kenacort-A 10) prior to balloon dilatation and balloon dilatation without any injection. The participating centres are tertiary (academic) hospitals that collaborate



For peer review only - http://bmjopen.bmj.com/site/about/guidelines.xhtml

within the European Reference Network on Inherited and Congenital Abnormalities (ERNICA, ern-ernica.eu) and that routinely provide care for children with OA.

**Patient and public involvement**

Parents of patients were involved in the end-stage of the design of the trial. We presented our plans for the trial to a DCEA meeting in which also representatives of the patients association VOKS (*Vereniging voor Ouderen en Kinderen met een Slokdarmafsluiting*) took part. They were invited to comment on the study design, intervention or time required to participate in this trial. Consensus was reached on the final design during this meeting. The patients association will not be involved in the recruitment and conduct of the trial. We will involve them in dissemination, however, by presenting the trial results at a members' day or in their monthly newsletter. As the VOKS is a member of the European Federation of Esophageal Atresia and Tracheoesophageal fistula Support Groups E.V. (EAT), patients and parents throughout Europe will be informed about the results.

**Participants**

OA can be a very heterogeneous disease. About 90% of the children with OA has type C.[5, 25] In order to make the two treatment groups as equal as possible, only children with OA type C who underwent surgery with primary anastomosis within the first days of life and who developed a recurrent oesophageal stricture will be eligible. Children will be included if they are ≥3 months old at the time of the intervention and in need of a third dilatation. Written parental informed consent will be obtained by the local principal investigator (PI) or another member of the local research team. Exclusion criteria are lack of parental consent or an impossibility – known from previous dilatations – to use an endoscope with a large enough diameter working-channel to pass the endoscopic injector.

**Sample size calculation**

The power calculation is based on a linear-by-linear chi-square association test comparing the total number of dilatations required within the study period (all strictures) and within a 28-days interval (refractory strictures) between the two treatment groups. The total number of dilatations will be categorized into the categories 3, 4, 5, 6, 7-10 and >10 dilatations; and a separate category for patients who are not dysphagia-free at the end of the follow-up period.

For this power calculation, we used data of the original dataset of our retrospective study in the Netherlands.[5] We selected patients from this dataset who underwent at least three dilatations with a 28 days interval (n=53). The in the retrospective study observed numbers of patients and the relative frequencies for each category are listed in Table 2. We assumed that the use of intralesional steroid injections combined with endoscopic dilatation will reduce the total number of dilatations by 50%. Note that this 50% reduction only applies to dilatations after the third dilatation, and therefore no change in the number



For peer review only

of dilatations is assumed for the first three dilatations. This assumption leads to a different distribution for the number of dilatations within the categories, which is shown as the assumed relative frequencies for the steroid group in the final column of Table 2. The details of the calculation are provided in Supplementary File 1.

In a simulation model, the required sample size to obtain a power of 80% (with a two-sided significance level of 0.05) was calculated as 52 patients per group, thus 104 in total. To account for the effects of dropout and missing data, we aim to include a total of 110 patients.

**Table 2: Assumed relative frequencies of the number of dilatations in the control and steroid groups.**

| Number of dilatations within 28 days interval | Observed number of patients (n=407)[5] | Relative frequencies control group | Assumed relative frequencies steroid group |
|---|---|---|---|
| 3 dilatations | 4 | 0.075 | 0.142 |
| 4 dilatations | 7 | 0.132 | 0.302 |
| 5 dilatations | 9 | 0.170 | 0.170 |
| 6 dilatations | 7 | 0.132 | 0.160 |
| 7-10 dilatations | 16 | 0.302 | 0.132 |
| >10 dilatations | 10 | 0.189 | 0.094 |
| Total (all numbers of dilatations combined) | 53 | 1.000 | 1.000 |

**Recruitment**

Patients will be recruited from hospitals in various European countries. Up till now, hospitals in Denmark, United Kingdom, Finland, France, Italy, and Sweden have agreed to participate. During the inclusion period it will remain possible for other centres to join. To achieve adequate participant enrolment, we have minimalized the exclusion criteria. Collaboration within ERNICA should make patient accrual achievable despite the rarity of the disease.

**Randomisation, blinding and treatment allocation**

Randomisation will be conducted via ALEA (FormsVision B.V. / ALEA Clinica B.V.), a validated software program. To achieve equal distribution of the intervention among the participating sites, block randomisation stratified per centre will be carried out. The software was prepared by an independent statistician who is not otherwise involved in the study. After inclusion, the local PI will enter the patient in ALEA and will thereupon receive an email stating the allocated treatment.

Randomization will be blinded for the radiologist who will review all oesophagrams. The control group will not receive sham treatment. The steroid that will be used, Kenacort-A 10 (see below), is a white suspension, which complicates creating a placebo. Adding excipients to normal saline is undesirable, considering the unknown effect on the healing process of the stricture. Moreover, it is deemed undesirable to inject an infant with a fluid with an unknown effect.



For peer review only - http://bmjopen.bmj.com/site/about/guidelines.xhtml

**Investigational product**

The intervention will be a one-time endoscopic injection of 0.25 mL Kenacort-A 10 (Bristol-Myers Squibb BV, Utrecht, the Netherlands) in each quadrant of the stricture prior to the third endoscopic dilatation. Thus, in total, 1 mL (10 mg/ml TAC, equals 12.5 mg prednisone) will be injected. During the study period, none of the patients will receive a second injection.

Kenacort-A 10 will be prepared, labelled and distributed by the trial pharmacy of the coordinating hospital. It will be delivered to the local pharmacies by courier, and the local pharmacy will deliver it to the operation room when needed. After the trial, a specific procedure for destruction of the remaining drugs is not needed; they can be disposed of locally.

**Patient timeline**

Figure 1 presents a flowchart for this study and the study procedures. The treating physician will decide on a third dilatation on the basis of the clinical signs of dysphagia and the findings on the oesophagram (thoracic x-ray with contrast, anterior-posterior and lateral). Clinical signs of dysphagia are defined as the inability to be fed age-appropriately. Findings on the oesophagram indicating a stricture are defined as a significant narrowing of the lumen, seen as a waist in the contrast on the x-ray. The treating physician can be a paediatric surgeon or paediatric gastroenterologist, depending on the local agreements in the different countries. After parental informed consent, the patient will be included and randomised to one of the study arms.

Prior to the balloon dilatation, an endoscopic needle (DVI-23-MH varices injector or equivalent) is pre-filled with 1 mL Kenacort-A 10 and passed through the endoscope. Under direct vision, 0.25 mL will be injected in each of the four quadrants of the circular stricture. After the injection needle has been retracted from the working channel and good visualization has been re-established, balloon dilatation will be performed up to the desired diameter. The balloon will remain insufflated for one minute.

During a follow up period of six months, patients will not receive any (additional) steroid injections, only balloon dilatations if needed. Meaning, patients in the steroid group will only receive one injection, and patients in the control group will receive no injections at all. After the study period has ended, treatment is again free of choice.

Two to three weeks after the dilatation procedure, a second oesophagram will be made in children in both study groups. All oesophagrams will be reviewed by one specialized paediatric radiologist of the coordinating hospital, who will determine the oesophageal diameter and stricture length. Earlier studies have proven that these measurements can be obtained from an oesophagram.[26] The parents will be informed about the results and the normal standard of care will be continued.

Each patient will be followed up for six months after the third dilatation, with recording of possible side effects, complications and additional dilatations. Length and weight will be measured at 2-3 weeks, 3 months and 6 months after the third dilatation. In the context of the cost-effectiveness analysis, the parents



For peer review only - http://bmjopen.bmj.com/site/about/guidelines.xhtml
For peer review only

will be asked to fill out a modified version of the iMTA Productivity Cost Questionnaire (iPCQ)[27] at 3 months after the third dilatation.

At evaluation at 6 months, we will collect a scalp hair sample from the child to determine long-term cortisone and cortisol levels.[28, 29] The hair locks will be stored for final batch analysis. Last, the parents will be asked to fill out the Montreal Feeding Scale (MFS)[30-32] and again the iPCQ. After this, the study period has ended.

**Outcome parameters**

The primary outcome parameter is the total number of dilatations required per patient with a 28 days interval between the dilatations during the study period of six months, which is defined as the period from the day of the third dilatation until six months later.

The secondary outcome parameters are:

1) Total number of dilatations within the study period, regardless of the interval.
2) Interval (in weeks) between the start of the study and the last dilatation procedure within the study period.
3) Scores on the MFS.
4) The change in maximal luminal diameter after the third dilatation relative to the diameter before the third dilatation: relative change in luminal diameter = (maximal diameter after – maximal diameter before) / maximal diameter before. The diameter will be measured at the narrowest point of the oesophagus.
5) The change in length of the oesophageal stricture after the third dilatation relative to the length before the third dilatation: relative change in stricture length = (stricture length after – stricture length before) / stricture length before. The length will be measured between the two points where the oesophageal diameter starts narrowing.
6) The use of co-medication (e.g. antacids) during the study period.
7) The mean hair cortisol levels in the first three months after the third dilatation. Cortisol levels will be adjusted for age and sex.
8) Delta length standard deviation scores (SDS) and delta weight SDS between the third dilatation (intervention) and 3 and 6 months after the third dilatation.
9) Total costs of the treatment, including medical and non-medical costs.
10) Incremental costs per refractory stricture prevented and incremental costs per additional dysphagia-free patient.

**Data collection**

All participating centres are familiar with the procedure of injecting Kenacort-A 10 in the lesion via an injection needle through the endoscope. However, to guarantee equality of the intervention, the relevant



For peer review only - http://bmjopen.bmj.com/site/about/guidelines.xhtml

practitioner(s) in all centres will be trained by the PI of the trial. Radiologic interobserver variability will be avoided by having all oesophagrams reviewed by one radiologist.

The internationally validated MFS[30-32] will be used to measure dysphagia. The MFS has often been used in previous research in children with OA. The scalp hair sample will be taken from the posterior vertex; cortisol levels will be determined with the liquid chromatography-tandem mass spectrometry (LC-MS/MS) method for quantification of steroids.[28]

The cost-effectiveness analysis will follow established methods for economic evaluations and costing studies in health care.[33, 34] Both medical and non-medical costs will be collected. Medical costs will include costs of surgeries (dilatations), steroid injections, hospital days (on the ward or the intensive care unit), medication (such as analgesics) and diagnostic radiography. Costs of health care provided by others than the participating centres (such as other hospitals or general practitioners) will be ignored in this study, as these are unlikely to be affected by the intervention. Non-medical costs will include costs of special diets, costs related to hospital visits and productivity losses related to both paid and unpaid work. The non-medical costs will be measured using the iPCQ[27], supplemented with additional questions on costs of special diets and costs related to the child's hospitalisation. The original iPCQ questions are validated in English. The complete questionnaire including the additional questions will be translated to the languages required for this trial using the forward-backward translation method, as will the MFS for the languages it has not been validated for.

**Statistical analysis**

Since a standardized treatment protocol will be adopted in all centres, statistical adjustment for centre effects is considered unnecessary.

The primary outcome parameter will be analysed with a linear-by-linear chi-square association test. The total number of dilatations required with a 28 days interval (i.e. refractory strictures) within the study period of 6 months per patient will be categorized and compared between treatment groups. In case of death during the follow-up period, the outcome will be set to the highest (i.e. most severe) category. In case of drop-out during the follow-up period due to other causes (e.g. emigration, withdrawal), the subject will be excluded from the study.

The analyses for the secondary study parameters are as follows:

1) The total number of dilatations required within the study period (regardless of the interval, i.e. all strictures) will be categorized and compared between treatment groups with a linear-by-linear chi-square association test.
2) The interval (in weeks) until the patient is dysphagia-free will be compared between groups with the log-rank test and with Cox proportional hazards regression, with adjustment for treatment group and factors such as age, sex, diagnostic information and described risk factors for stricture formation like



For peer review only - http://bmjopen.bmj.com/site/about/guidelines.xhtml

anastomotic leaking and thoracoscopic repair.[5] Patients who do not become dysphagia-free during follow-up are treated as censored at the end of the follow-up period.

3) The scores on the MFS (reflecting the level of dysphagia and the eating behaviour) will be compared between study groups with a Mann-Whitney test.

4) The relative change in the oesophageal diameter and that in the length of the oesophageal stricture will be compared between study groups with an analysis of covariance model. The dependent variables in this model will be the log-transformed oesophageal diameter and length after third dilatation, and the independent variables the treatment group and the log-transformed oesophageal diameter and length before third dilatation.

6) The effect of co-medication on the primary study outcome will be assessed using a stratified Mann-Whitney test with stratification for the treatment group.

7) The mean cortisol level over the first three months after the third dilatation will be compared between study groups with a linear regression model with adjustment for age and sex.

No missing data are expected for the independent variables in the analysis of covariance models and the Cox proportional hazards regression models. In case of missing data for any of the outcomes, a complete case analysis (i.e. exclusion of the subjects who dropped out during the follow-up period) will be performed for the corresponding outcome.

**Cost-effectiveness analysis**

All medical and non-medical costs will be summated for each individual patient. Regarding the patient outcomes, the number of refractory strictures prevented and the number of dysphagia-free patients will be considered. Incremental cost-effectiveness ratios will be calculated, expressed as incremental costs per refractory stricture prevented and incremental costs per additional dysphagia-free patient. Analysis of uncertainty will be illustrated through cost-effectiveness planes (via bootstrapping).

As this RCT is done in an international setting, it must be recognized that the results may differ between countries because health care systems, treatment patterns and prices may vary. Therefore, country-level information will be collected. Data (especially resource quantities and cost prices) will be collected in all countries, and results will also be reported for each country separately. Next, a pooled summary calculation of the intervention's cost-effectiveness will be made, converting all costs into a common currency base (i.e. Euros).

**Adverse events and auditing**

Adverse events will be handled according to the guidelines of the IRB of the Erasmus Medical Centre. All adverse events will be registered during the study. Serious adverse events will be reported to the sponsor immediately and registered appropriately within 24 hours.



For peer review only - http://bmjopen.bmj.com/site/about/guidelines.xhtml

All participating sites will be audited once a year with monitoring of patient recruitment, source data verification, drug accountability and sample storage. The auditor will be independent and not involved in the study.

**Benefits and risk assessment**

The risks and burden associated with this study are minimal. Potential complications of oesophageal steroid injections include adrenal suppression, perforation, intramural infection, candida infection, mediastinitis and pleural effusion.[35] However, in previous studies no adverse events have been reported in relation to the steroid injections (see Table 1). Additionally, Kenacort-A 10 is a slow-release medicine and therefore a gradual exposure. This implies that the likelihood of acute exposure to a high dose of steroids will be minimal.

The burden of filling out the questionnaires and taking a hair sample are negligible. Filling out the MFS and the iPCQ will take maximally 30 minutes. All participants will undergo one extra oesophagram after the third dilatation procedure. The potential reduction in number of anaesthetic procedures needed for dilatations outweighs the burden and the radiation exposure of this oesophagram. Potential benefits of intralesional steroid injections are fewer dilatation procedures needed, with concomitant fewer anaesthetic procedures and hospital admissions, and less risk for perforation.

**Data management**

All data will be handled confidentially and anonymously using OpenClinica V3.12.2 (OpenClinica LLC, USA) for data collection. The questionnaires will be conducted through an online survey using LimeSurvey (LimeSurvey GmbH, Germany) and GemsTracker (Erasmus MC, Rotterdam, the Netherlands). All patient data will be coded using a subject identification code list. The local PI safeguards the key to the code; the sponsor will have access to these codes. The local PI will only have access to the data of patients of their own centre; the sponsor will have access to the final trial dataset. All has been stated in a clinical trial site agreement, signed by all participating sites.

# ETHICS AND DISSEMINATION

This study protocol was approved by the Institutional Review Board (IRB) of the Erasmus Medical Centre (MEC-2018-1586/NL65364.078.18). In case of any modifications of the protocol, a formal amendment will be submitted to the IRB. Approved changes will be communicated to all relevant parties according to the rules of the IRB. The protocol has currently been submitted for local ethical approval in Amsterdam (the Netherlands), Odense (Denmark), Copenhagen (Denmark), Stockholm (Sweden), Rome (Italy) and Padua (Italy). Nijmegen (the Netherlands) and Helsinki (Finland) have already obtained local ethical approval and joined the study. To guarantee the respect of ethical rules and standard of care in all participating centres,



For peer review only - http://bmjopen.bmj.com/site/about/guidelines.xhtml

the protocol was reviewed by the two chairs of the work package on OA within ERNICA. The informed consent and assent process of this trial is in line with the Good Clinical Practice guideline.[36]

Furthermore, we involved external experts in the form of a Data Safety Monitoring Board (DSMB). These experts are a paediatric surgeon, a pharmacologist and a statistician. All experts are independent of the sponsor and therefore competing interests will be avoided. The DSMB will monitor the safety of the study subjects and data. The board will meet at least three times: within one year of recruitment commencing, at the time of the planned interim analyses at 50% (n=55) of enrolment and at the conclusion of the trial.

The results of this trial will be published in an international peer-reviewed scientific journal, within one year after the end of the follow-up period of the last included patient. In addition, we aim to present the results at several international conferences, to inform health care professionals worldwide.

## TRIAL STATUS

The study has started in Rotterdam (the Netherlands) in February 2019, after ethical approval had been obtained. The first patient is included. Nijmegen (the Netherlands) has joined the study in October 2019 and Helsinki (Finland) in December 2019. At this moment, Amsterdam (the Netherlands), Odense (Denmark), Copenhagen (Denmark), Stockholm (Sweden), Rome (Italy) and Padua (Italy) are still waiting for local ethical approval. We expect to start the study in these sites the latest in 2020.

Considering the rarity of the disease, we expect to complete the inclusions and finish data collection for this study in five years.

For peer review only

## ACKNOWLEDGEMENTS

The author thank Benno Ure, paediatric surgeon and head of department of Paediatric Surgery of Hannover Medical School, and Frédéric Gottrand, paediatrician at the department of Paediatric Gastroenterology of University Hospital of Lille, for their input during the development of the trial protocol. The authors thank Joke Dunk, research nurse at the Erasmus University Medical Centre in Rotterdam, for her help with the implementation of the trial. Ko Hagoort provided editorial advice. At last, we thank all hospitals involved in ERNICA and the VOKS for their input and collaboration.

## AUTHORS CONTRIBUTIONS

RW is the initiator of the study. CK (PhD candidate), JV (paediatric surgeon), HIJ (paediatrician), KA (paediatric clinical pharmacologist), MS (gastroenterologist), MP (health economist), JR (statistician), EA (paediatric endocrinologist) and RW (paediatric surgeon) contributed in writing and designing of the study protocol. JV (principal investigator) and RW (project leader) will supervise the study. CK will coordinate the study and be responsible for data acquisition. CK, JV and JR will carry out the analyses. All authors have read and approved the final version of the study protocol and manuscript.

## FUNDING STATEMENT

This research received no specific grant from any funding agency in the public, commercial or not-for-profit sectors.

## COMPETING INTEREST STATEMENT

None declared.

## LICENSE STATEMENT

I, the Submitting Author has the right to grant and does grant on behalf of all authors of the Work (as defined in the below author licence), an exclusive licence and/or a non-exclusive licence for contributions from authors who are: i) UK Crown employees; ii) where BMJ has agreed a CC-BY licence shall apply, and/or iii) in accordance with the terms applicable for US Federal Government officers or employees acting as part of their official duties; on a worldwide, perpetual, irrevocable, royalty-free basis to BMJ Publishing Group Ltd ("BMJ") its licensees and where the relevant Journal is co-owned by BMJ to the co-owners of the Journal, to publish the Work in BMJ Open and any other BMJ products and to exploit all rights, as set out in our licence.

The Submitting Author accepts and understands that any supply made under these terms is made by BMJ to the Submitting Author unless you are acting as an employee on behalf of your employer or a postgraduate



For peer review only - http://bmjopen.bmj.com/site/about/guidelines.xhtml

student of an affiliated institution which is paying any applicable article publishing charge ("APC") for Open Access articles. Where the Submitting Author wishes to make the Work available on an Open Access basis (and intends to pay the relevant APC), the terms of reuse of such Open Access shall be governed by a Creative Commons licence – details of these licences and which Creative Commons licence will apply to this Work are set out in our licence referred to above.

## DATA AVAILABILIY STATEMENT

The data that support the findings of this trial will be available from the corresponding author upon reasonable request once the results are published.

For peer review only

# REFERENCES


1. Oddsberg J, Lu Y, Lagergren J. Aspects of esophageal atresia in a population-based setting: incidence, mortality, and cancer risk. *Pediatr Surg Int* 2012;28(3):249-57.
2. Pedersen RN, Calzolari E, Husby S, et al. Oesophageal atresia: prevalence, prenatal diagnosis and associated anomalies in 23 European regions. *Arch Dis Child* 2012;97(3):227-32.
3. Wang B, Tashiro J, Allan BJ, et al. A nationwide analysis of clinical outcomes among newborns with esophageal atresia and tracheoesophageal fistulas in the United States. *J Surg Res* 2014;190(2):604-12.
4. Sulkowski JP, Cooper JN, Lopez JJ, et al. Morbidity and mortality in patients with esophageal atresia. *Surgery* 2014;156(2):483-91.
5. Vergouwe FWT, Vlot J, H IJ, et al. Risk factors for refractory anastomotic strictures after oesophageal atresia repair: a multicentre study. *Arch Dis Child* 2018.
6. Krishnan U, Mousa H, Dall'Oglio L, et al. ESPGHAN-NASPGHAN Guidelines for the Evaluation and Treatment of Gastrointestinal and Nutritional Complications in Children With Esophageal Atresia-Tracheoesophageal Fistula. *J Pediatr Gastroenterol Nutr* 2016;63(5):550-70.
7. Tringali A, Thomson M, Dumonceau JM, et al. Pediatric gastrointestinal endoscopy: European Society of Gastrointestinal Endoscopy (ESGE) and European Society for Paediatric Gastroenterology Hepatology and Nutrition (ESPGHAN) Guideline Executive summary. *Endoscopy* 2017;49(1):83-91.
8. Ten Kate CA, Vlot J, Sloots CEJ, et al. The effect of intralesional steroid injections on esophageal strictures and the child as whole: a case series. *J Pediatr Surg* 2019.
9. Holder TM, Ashcraft KW, Leape L. The treatment of patients with esophageal strictures by local steroid injections. *J Pediatr Surg* 1969;4(6):646-53.
10. Zein NN, Greseth JM, Perrault J. Endoscopic intralesional steroid injections in the management of refractory esophageal strictures. *Gastrointest Endosc* 1995;41(6):596-8.
11. Mendelsohn HJ, Maloney WH. The treatment of benign strictures of the esophagus with cortisone injection. *Ann Otol Rhinol Laryngol* 1970;79(5):900-4.
12. Gandhi RP, Cooper A, Barlow BA. Successful management of esophageal strictures without resection or replacement. *J Pediatr Surg* 1989;24(8):745-9; discussion 49-50.
13. Kirsch M, Blue M, Desai RK, et al. Intralesional steroid injections for peptic esophageal strictures. *Gastrointest Endosc* 1991;37(2):180-2.
14. Kochhar R, Ray JD, Sriram PV, et al. Intralesional steroids augment the effects of endoscopic dilation in corrosive esophageal strictures. *Gastrointest Endosc* 1999;49(4 Pt 1):509-13.
15. Camargo MA, Lopes LR, Grangeia Tde A, et al. [Use of corticosteroids after esophageal dilations on patients with corrosive stenosis: prospective, randomized and double-blind study]


For peer review only



For peer review only - http://bmjopen.bmj.com/site/about/guidelines.xhtml

O uso de corticoesteroides apos dilata o esofagica em pacientes portadores de estenose por substancias corrosivas: estudo prospectivo, randomizado e duplo-cego. *Rev Assoc Med Bras (1992)* 2003;49(3):286-92.

16. Ramage JI, Jr., Rumalla A, Baron TH, et al. A prospective, randomized, double-blind, placebo-controlled trial of endoscopic steroid injection therapy for recalcitrant esophageal peptic strictures. *Am J Gastroenterol* 2005;100(11):2419-25.
17. Hirdes MM, van Hooft JE, Koornstra JJ, et al. Endoscopic corticosteroid injections do not reduce dysphagia after endoscopic dilation therapy in patients with benign esophagogastric anastomotic strictures. *Clin Gastroenterol Hepatol* 2013;11(7):795-801 e1.
18. Pereira-Lima JC, Lemos Bonotto M, Hahn GD, et al. A prospective randomized trial of intralesional triamcinolone injections after endoscopic dilation for complex esophagogastric anastomotic strictures: steroid injection after endoscopic dilation. *Surg Endosc* 2015;29(5):1156-60.
19. Kochhar R, Makharia GK. Usefulness of intralesional triamcinolone in treatment of benign esophageal strictures. *Gastrointest Endosc* 2002;56(6):829-34.
20. Nijhawan S, Udawat HP, Nagar P. Aggressive bougie dilatation and intralesional steroids is effective in refractory benign esophageal strictures secondary to corrosive ingestion. *Dis Esophagus* 2016;29(8):1027-31.
21. Divarci E, Celtik U, Dokumcu Z, et al. The Efficacy of Intralesional Steroid Injection in the Treatment of Corrosive Esophageal Strictures in Children. *Surg Laparosc Endosc Percutan Tech* 2016;26(6):e122-e25.
22. Cakmak M, Boybeyi O, Gollu G, et al. Endoscopic balloon dilatation of benign esophageal strictures in childhood: a 15-year experience. *Dis Esophagus* 2016;29(2):179-84.
23. Ashcraft KW, Holder TM. The expeimental treatment of esophageal strictures by intralesional steroid injections. *J Thorac Cardiovasc Surg* 1969;58(5):685-91 passim.
24. Ketchum LD, Smith J, Robinson DW, et al. The treatment of hypertrophic scar, keloid and scar contracture by triamcinolone acetonide. *Plast Reconstr Surg* 1966;38(3):209-18.
25. Gross RE. Atresia of the esophagus. *Am J Dis Child* 1947;74(3):369.
26. Sun LY, Laberge JM, Yousef Y, et al. The Esophageal Anastomotic Stricture Index (EASI) for the management of esophageal atresia. *J Pediatr Surg* 2015;50(1):107-10.
27. Bouwmans C, Krol M, Severens H, et al. The iMTA Productivity Cost Questionnaire: A Standardized Instrument for Measuring and Valuing Health-Related Productivity Losses. *Value Health* 2015;18(6):753-8.
28. Noppe G, de Rijke YB, Dorst K, et al. LC-MS/MS-based method for long-term steroid profiling in human scalp hair. *Clin Endocrinol (Oxf)* 2015;83(2):162-6.
29. Wester VL, Noppe G, Savas M, et al. Hair analysis reveals subtle HPA axis suppression associated with use of local corticosteroids: The Lifelines cohort study. *Psychoneuroendocrinology* 2017;80:1-6.

For peer review only



For peer review only - http://bmjopen.bmj.com/site/about/guidelines.xhtml

30. Baird R, Levesque D, Birnbaum R, et al. A pilot investigation of feeding problems in children with esophageal atresia. *Dis Esophagus* 2015;28(3):224-8.
31. Ramsay M, Martel C, Porporino M, et al. The Montreal Children's Hospital Feeding Scale: A brief bilingual screening tool for identifying feeding problems. *Paediatr Child Health* 2011;16(3):147-e17.
32. Van Dijk M, Timmerman M, Martel C, et al. Towards the development of a Dutch screening instrument for the detection of feeding problems in young children. *Netherlands Journal of Psychology* 2011;66:112-19.
33. National Health Institute Diemen. Guidelines for economic evaluations of health care [in Dutch].
34. National Health Care Institute Diemen. Manual for costing research. Methods and reference prices for economic evaluations in health care. 2015 Updated edition [in Dutch]. .
35. Kochhar R, Poornachandra KS. Intralesional steroid injection therapy in the management of resistant gastrointestinal strictures. *World J Gastrointest Endosc* 2010;2(2):61-8.
36. ICH Expert Working Group. Guideline for Good Clinical Practice. 1996.

For peer review only

# FIGURE LEGENDS

**Figure 1: Flowchart of the study design**

OA = oesophageal atresia, AP = anterior-posterior, iPCQ = iMTA Productivity Cost Questionnaire, MFS = Montreal Feeding Scale. **Bold with underline** = study procedures, the rest is standard of care.

For peer review only



For peer review only - http://bmjopen.bmj.com/site/about/guidelines.xhtml

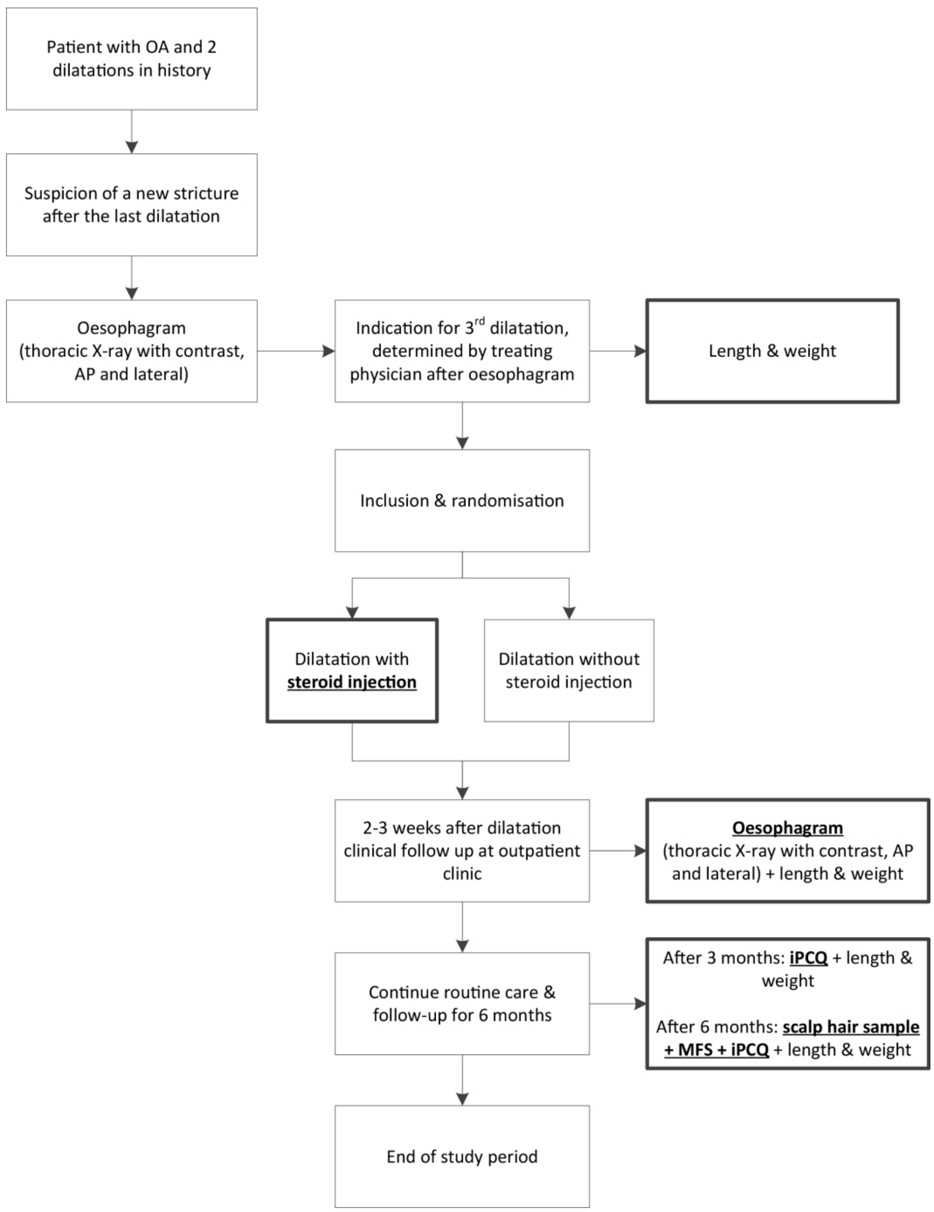


Figure 1: Flowchart of the study design
OA = oesophageal atresia, AP = anterior-posterior, iPCQ = iMTA Productivity Cost Questionnaire, MFS = Montreal Feeding Scale. Bold with underline = study procedures, the rest is standard of care.

# SUPPLEMENTARY FILE 1

To calculate the distribution of the number of dilatations in the steroid group, we assumed the following:

- The first 3 dilatations are unaffected by the intervention; thus the 4 patients with 3 dilatations in the observed data would still have 3 dilatations with steroid treatment.
- The 7 patients with 4 dilatations in the observed data would, if steroid treatment were applied, be equally divided over the categories 3 dilatations and 4 dilatations (3.5 patients in each category).
- The 9 patients with 5 dilatations in the observed data would, if steroid treatment were applied, have 4 dilatations.
- The 7 patients with 6 dilatations in the observed data would, if steroid treatment were applied, be equally divided over the categories 4 dilatations and 5 dilatations (3.5 patients in each category).
- The 16 patients with 7-10 dilatations in the observed data would, if steroid treatment were applied, be divided over the categories 5, 6 and 7-10 dilatations (patients with 7 dilatations would have 5 dilatations, patients with 8 dilatations would have 5 or 6 dilatations, patients with 9 dilatations would have 6 dilatations and patients with 10 dilatations would have 6 or 7 dilatations).
- The 10 patients with >10 dilatations in the observed data would, if steroid treatment were applied, be divided over the categories 7-10 and >10 dilatations (5 patients in each category).

These assumptions lead to the predicted numbers of patients shown in Supplementary Table 1. The relative frequency distribution in the steroid group is then calculated by dividing the predicted numbers of patients by the total number of 53 patients.

| Number of dilatations within 28 days interval | Observed number of patients (n=407) [1] | Predicted number of patients with steroid treatment | Relative frequencies control group | Assumed relative frequencies steroid group |
|---|---|---|---|---|
| 3 dilatations | 4 | 7.5 (4+3.5) | 0.075 | 0.142 |
| 4 dilatations | 7 | 16 (3.5+9+3.5) | 0.132 | 0.302 |
| 5 dilatations | 9 | 9 (3.5+2+3.5) | 0.170 | 0.170 |
| 6 dilatations | 7 | 8.5 (3.5+3+2) | 0.132 | 0.160 |
| 7-10 dilatations* | 16 | 7 (2+5) | 0.302 | 0.132 |
| >10 dilatations* | 10 | 5 (5) | 0.189 | 0.094 |
| Total (all numbers of dilatations combined) | 53 | 53 | 1.000 | 1.000 |

**Supplementary Table 1:** Assumed relative frequencies of the number of dilatations in the control and steroid groups, including the calculated predicted number of patients with steroid treatment. *See Supplementary Table 2 for the exact number of dilatations within these categories.

For peer review only

| **Number of dilatations within 28 days interval** | **Observed number of patients (n=407)[1]** |
|---|---|
| 3 dilatations | 4 |
| 4 dilatations | 7 |
| 5 dilatations | 9 |
| 6 dilatations | 7 |
| 7 dilatations | 2 |
| 8 dilatations | 7 |
| 9 dilatations | 3 |
| 10 dilatations | 4 |
| 12 dilatations | 2 |
| 13 dilatations | 2 |
| 15 dilatations | 1 |
| 18 dilatations | 2 |
| 24 dilatations | 1 |
| 30 dilatations | 1 |
| 34 dilatations | 1 |
| Total (all numbers of dilatations combined) | 53 |

**Supplementary Table 2:** *Exact* number of dilatations as extracted from the *original dataset* of the retrospective study in the Netherlands.[1]

1. Vergouwe FWT, Vlot J, H IJ, et al. Risk factors for refractory anastomotic strictures after oesophageal atresia repair: a multicentre study. *Arch Dis Child* 2018.

For peer review only

# Reporting checklist for protocol of a clinical trial.

Based on the SPIRIT guidelines.

## Instructions to authors

Complete this checklist by entering the page numbers from your manuscript where readers will find each of the items listed below.

Your article may not currently address all the items on the checklist. Please modify your text to include the missing information. If you are certain that an item does not apply, please write "n/a" and provide a short explanation.

Upload your completed checklist as an extra file when you submit to a journal.

In your methods section, say that you used the SPIRITreporting guidelines, and cite them as:

Chan A-W, Tetzlaff JM, Altman DG, Laupacis A, Gøtzsche PC, Krleža-Jerić K, Hróbjartsson A, Mann H, Dickersin K, Berlin J, Doré C, Parulekar W, Summerskill W, Groves T, Schulz K, Sox H, Rockhold FW, Rennie D, Moher D. SPIRIT 2013 Statement: Defining standard protocol items for clinical trials. Ann Intern Med. 2013;158(3):200-207

For peer review only

| | | Reporting Item | Page Number |
|---|---|---|---|
| **Administrative information** | | | |
| Title | #1 | Descriptive title identifying the study design, population, interventions, and, if applicable, trial acronym | 1 |
| Trial registration | #2a | Trial identifier and registry name. If not yet registered, name of intended registry | 2 |
| Trial registration: data set | #2b | All items from the World Health Organization Trial Registration Data Set | 2 |
| Protocol version | #3 | Date and version identifier | 2 |
| Funding | #4 | Sources and types of financial, material, and other support | 15 |
| Roles and responsibilities: contributorship | #5a | Names, affiliations, and roles of protocol contributors | 15 |

| | | | |
|---|---|---|---|
| Roles and responsibilities: sponsor contact information | #5b | Name and contact information for the trial sponsor | 1 |
| Roles and responsibilities: sponsor and funder | #5c | Role of study sponsor and funders, if any, in study design; collection, management, analysis, and interpretation of data; writing of the report; and the decision to submit the report for publication, including whether they will have ultimate authority over any of these activities | 15 |
| Roles and responsibilities: committees | #5d | Composition, roles, and responsibilities of the coordinating centre, steering committee, endpoint adjudication committee, data management team, and other individuals or groups overseeing the trial, if applicable (see Item 21a for data monitoring committee) | 15 |
| **Introduction** | | | |
| Background and rationale | #6a | Description of research question and justification for undertaking the trial, including summary of relevant studies (published and unpublished) examining benefits and harms for each intervention | 4-6 |
| Background and rationale: choice of comparators | #6b | Explanation for choice of comparators | 4-6 |
| Objectives | #7 | Specific objectives or hypotheses | 6 |
| Trial design | #8 | Description of trial design including type of trial (eg, parallel group, crossover, factorial, single group), allocation ratio, and framework (eg, superiority, equivalence, non-inferiority, exploratory) | 6-7 |
| **Methods: Participants, interventions, and outcomes** | | | |
| Study setting | #9 | Description of study settings (eg, community clinic, academic hospital) and list of countries where data will be collected. Reference to where list of study sites can be obtained | 6-7 |
| Eligibility criteria | #10 | Inclusion and exclusion criteria for participants. If applicable, eligibility criteria for study centres and individuals who will | 7 |

For peer review only

| | | | |
|---|---|---|---|
| | | perform the interventions (eg, surgeons, psychotherapists) | |
| Interventions: description | #11a | Interventions for each group with sufficient detail to allow replication, including how and when they will be administered | 9 |
| Interventions: modifications | #11b | Criteria for discontinuing or modifying allocated interventions for a given trial participant (eg, drug dose change in response to harms, participant request, or improving / worsening disease) | N/A |
| Interventions: adherance | #11c | Strategies to improve adherence to intervention protocols, and any procedures for monitoring adherence (eg, drug tablet return; laboratory tests) | N/A |
| Interventions: concomitant care | #11d | Relevant concomitant care and interventions that are permitted or prohibited during the trial | 9 |
| Outcomes | #12 | Primary, secondary, and other outcomes, including the specific measurement variable (eg, systolic blood pressure), analysis metric (eg, change from baseline, final value, time to event), method of aggregation (eg, median, proportion), and time point for each outcome. Explanation of the clinical relevance of chosen efficacy and harm outcomes is strongly recommended | 10 |
| Participant timeline | #13 | Time schedule of enrolment, interventions (including any run-ins and washouts), assessments, and visits for participants. A schematic diagram is highly recommended (see Figure) | 9 |
| Sample size | #14 | Estimated number of participants needed to achieve study objectives and how it was determined, including clinical and statistical assumptions supporting any sample size calculations | 7-8 |
| Recruitment | #15 | Strategies for achieving adequate participant enrolment to reach target sample size | 8 |
| **Methods: Assignment of interventions (for controlled trials)** | | | |
| Allocation: sequence generation | #16a | Method of generating the allocation sequence (eg, computer-generated random numbers), and list of any factors for stratification. To reduce predictability of a random sequence, details of any planned restriction (eg, blocking) should be provided in a separate document that is unavailable to those who enrol participants or assign interventions | 8 |

For peer review only

| | | | |
|---|---|---|---|
| Allocation concealment mechanism | #16b | Mechanism of implementing the allocation sequence (eg, central telephone; sequentially numbered, opaque, sealed envelopes), describing any steps to conceal the sequence until interventions are assigned | 8 |
| Allocation: implementation | #16c | Who will generate the allocation sequence, who will enrol participants, and who will assign participants to interventions | 8 |
| Blinding (masking) | #17a | Who will be blinded after assignment to interventions (eg, trial participants, care providers, outcome assessors, data analysts), and how | 8 |
| Blinding (masking): emergency unblinding | #17b | If blinded, circumstances under which unblinding is permissible, and procedure for revealing a participant's allocated intervention during the trial | N/A |
| **Methods: Data collection, management, and analysis** | | | |
| Data collection plan | #18a | Plans for assessment and collection of outcome, baseline, and other trial data, including any related processes to promote data quality (eg, duplicate measurements, training of assessors) and a description of study instruments (eg, questionnaires, laboratory tests) along with their reliability and validity, if known. Reference to where data collection forms can be found, if not in the protocol | 10-11 |
| Data collection plan: retention | #18b | Plans to promote participant retention and complete follow-up, including list of any outcome data to be collected for participants who discontinue or deviate from intervention protocols | 10-11 |
| Data management | #19 | Plans for data entry, coding, security, and storage, including any related processes to promote data quality (eg, double data entry; range checks for data values). Reference to where details of data management procedures can be found, if not in the protocol | 10-11 |
| Statistics: outcomes | #20a | Statistical methods for analysing primary and secondary outcomes. Reference to where other details of the statistical analysis plan can be found, if not in the protocol | 11-12 |
| Statistics: additional analyses | #20b | Methods for any additional analyses (eg, subgroup and adjusted analyses) | 11-12 |

For peer review only

| | | | |
|---|---|---|---|
| Statistics: analysis population and missing data | #20c | Definition of analysis population relating to protocol non-adherence (eg, as randomised analysis), and any statistical methods to handle missing data (eg, multiple imputation) | 11-12 |
| **Methods: Monitoring** | | | |
| Data monitoring: formal committee | #21a | Composition of data monitoring committee (DMC); summary of its role and reporting structure; statement of whether it is independent from the sponsor and competing interests; and reference to where further details about its charter can be found, if not in the protocol. Alternatively, an explanation of why a DMC is not needed | 13 |
| Data monitoring: interim analysis | #21b | Description of any interim analyses and stopping guidelines, including who will have access to these interim results and make the final decision to terminate the trial | 13 |
| Harms | #22 | Plans for collecting, assessing, reporting, and managing solicited and spontaneously reported adverse events and other unintended effects of trial interventions or trial conduct | 13 |
| Auditing | #23 | Frequency and procedures for auditing trial conduct, if any, and whether the process will be independent from investigators and the sponsor | 13 |
| **Ethics and dissemination** | | | |
| Research ethics approval | #24 | Plans for seeking research ethics committee / institutional review board (REC / IRB) approval | 12 |
| Protocol amendments | #25 | Plans for communicating important protocol modifications (eg, changes to eligibility criteria, outcomes, analyses) to relevant parties (eg, investigators, REC / IRBs, trial participants, trial registries, journals, regulators) | 12 |
| Consent or assent | #26a | Who will obtain informed consent or assent from potential trial participants or authorised surrogates, and how (see Item 32) | 7 |
| Consent or assent: ancillary studies | #26b | Additional consent provisions for collection and use of participant data and biological specimens in ancillary studies, if applicable | N/A |
| Confidentiality | #27 | How personal information about potential and enrolled participants will be collected, shared, and maintained in order to protect confidentiality before, during, and after the trial | 10-11 |

For peer review only

| | | | |
|---|---|---|---|
| Declaration of interests | #28 | Financial and other competing interests for principal investigators for the overall trial and each study site | 15 |
| Data access | #29 | Statement of who will have access to the final trial dataset, and disclosure of contractual agreements that limit such access for investigators | 10-11 |
| Ancillary and post trial care | #30 | Provisions, if any, for ancillary and post-trial care, and for compensation to those who suffer harm from trial participation | N/A |
| Dissemination policy: trial results | #31a | Plans for investigators and sponsor to communicate trial results to participants, healthcare professionals, the public, and other relevant groups (eg, via publication, reporting in results databases, or other data sharing arrangements), including any publication restrictions | 14 |
| Dissemination policy: authorship | #31b | Authorship eligibility guidelines and any intended use of professional writers | 14 |
| Dissemination policy: reproducible research | #31c | Plans, if any, for granting public access to the full protocol, participant-level dataset, and statistical code | 14 |
| **Appendices** | | | |
| Informed consent materials | #32 | Model consent form and other related documentation given to participants and authorised surrogates | N/A |
| Biological specimens | #33 | Plans for collection, laboratory evaluation, and storage of biological specimens for genetic or molecular analysis in the current trial and for future use in ancillary studies, if applicable | N/A |

The SPIRIT checklist is distributed under the terms of the Creative Commons Attribution License CC-BY-ND 3.0. This checklist was completed on 15. July 2019 using https://www.goodreports.org/, a tool made by the EQUATOR Network in collaboration with Penelope.ai

For peer review only
